# Effect of 1-aminocyclopropane-1-carboxylic acid accumulation on *Verticillium dahliae* infection of upland cotton

**DOI:** 10.1186/s12870-022-03774-8

**Published:** 2022-08-03

**Authors:** Ming-zhu Jia, Zhi-fang Li, Shuan Han, Song Wang, Jing Jiang

**Affiliations:** State Key Laboratory of Cotton Biology, State Key Laboratory of Crop Stress Adaptation and Improvement, College of Life Sciences, Henan University, Jinming Street, Kaifeng, 475004 Henan Province China

**Keywords:** Cotton, *Verticillium dahliae*, *GhACS2*, *GhACS6*, ACC, Salicylic acid

## Abstract

**Background:**

Verticillium wilt of cotton is a serious disease caused by the infection of soil borne fungus *Verticillium dahliae* Kleb, and the infection mechanisms may involve the regulation of phytohormone ethylene. The precursor of ethylene biosynthesis is 1-aminocyclopropane-1-carboxylic acid (ACC), whose biosynthesis *in vivo* depends on activation of ACC synthase (ACS). Here, we investigated how ACS activation and ACC accumulation affected the infection of *V. dahliae* strain *Vd991* on cotton (*Gossypium hirsutum* L.) cultivar YZ1.

**Results:**

Preliminary observations indicated that ACC applications reduced the disease incidence, disease index and stem vascular browning by impeding fungal biomass accumulation. Transcriptome and qRT-PCR data disclosed that *Vd991* induced *GhACS2* and *GhACS6* expression. *GhACS2*- or *GhACS6*-overexpressing transgenic YZ1 lines were generated, respectively. In a *Verticillium* disease nursery with about 50 microsclerotia per gram of soil, these ACC-accumulated plants showed decreased disease indexes, stem fungal biomasses and vascular browning. More importantly, these transgenic plants decreased the green fluorescent protein-marked *Vd991* colonization and diffusion in root tissues. Further, either ACC treatment or ACC-accumulating cotton plants activated salicylic acid (SA)-dependent resistance responses.

**Conclusions:**

The *GhACS2*- and *GhACS6*-dependent ACC accumulations enhanced the resistance of cotton to *V. dahliae* in a SA-dependent manner, and this lays a foundation for cotton resistance breeding.

**Supplementary Information:**

The online version contains supplementary material available at 10.1186/s12870-022-03774-8.

## Background

Cotton is an important crop used for the production of fibers and oil worldwide. Cotton plants are often infected by various microbial pathogens, such as fungi and bacteria. Infection with the soil-borne fungus *Verticillium dahliae* Kleb. results in the vascular disease Verticillium wilt of cotton in the cultivated areas. Unfortunately, the widely cultivated cotton *Gossypium hirsutum* (*G. hirsutum*) lacks resistance to *V. dahliae*; therefore, serious infections are disastrous to cotton production [1].

The mechanisms by which *V. dahliae* invades host plant responses have been investigated. The fungus remains dormant in soil in the form of microsclerotia until suitable germination conditions occur [2]. The germinated microsclerotia produce hyphae, and the latter penetrate the plant root epidermis, cortex, endothelium and xylem, where they propagate and stimulate adjacent parenchymal cells to block the vessels and impair water and nutrient flux through the roots and shoots [2–4]. Although *V. dahliae* first infects the roots, Verticillium wilt disease occurs in shoots, and the typical disease symptoms include leaf wilting and necrosis, vascular bundle yellowing and browning, boll abscission and even plant death [1, 4, 5]. However, the regulatory mechanisms responsible for the spread of *V. dahliae* from root to shoot still need clarification.

In response to a *V. dahliae* infection, plants trigger resistance responses in a plant hormone-dependent manner. Among the studied plant hormones in their defense systems, the roles of salicylic acid (SA) and ethylene have been widely investigated [1, 2]. *Verticillium dahliae* infections enhance SA biosynthesis by increasing the expression of enhanced disease susceptibility 1 (*EDS1*), phytoalexin-deficient 4 (*PAD4*) and isochorismate synthase 1 (*ICS1*) genes [6]. The SA directly contributes to cotton resistance by activating the nonexpressor of pathogenesis-related protein 1 (*NPR1*) and pathogenesis-related genes (*PR*s), such as *PR1* and *PR5* [1, 7]. In contrast to SA, the role of ethylene in disease resistance responses is complicated [8]. Ethylene may aid the microbial pathogenecity during *V. dahliae* infection of plants [4, 9], but the relationship between ethylene production and host resistance has not been established.

Ethylene biosynthesis is the basis of its function. In plants, ethylene biosynthesis begins with S-adenosylmethionine, which is first broken down into a non-canonical amino acid known as 1-aminocyclopropane-1-carboxylic acid (ACC) by ACC synthase (ACS), and ACC is subsequently converted into ethylene by ACC oxidase [10]. Plant ACS enzymes are encoded by a multi-gene family [11], and the activities of ACS members are unique, overlapping and spatiotemporally specific [12, 13]. ACS2- and ACS6-generated ACC accumulations possess signaling roles in plant defenses beyond their functions in ethylene biosynthesis in *Arabidopsis* defenses against *V. dahliae* [4]. However, there is still a lack of evidence to explain how ACC production regulates the susceptibility and disease resistance of cotton to *V. dahliae*.

Here, we determined whether and how ACC production is involved in the susceptibility and disease resistance of upland cotton upon *V. dahliae* infection. Our observations indicated that inoculation with *Vd991* significantly increased the *GhACS2* and *GhACS6* expression levels. The transgenic *GhACS2-* and *GhACS6-*overexpressing lines significantly increased ACC accumulation, and reduced colonization and diffusion of *V. dahliae* in roots of cotton plants. Monitoring of the colonization dynamics using green fluorescent protein (GFP)-labeled *V. dahliae* indicated that ACC treatments or ACC-accumulated plants impeded *V. dahliae* spread from roots to stems. The improvement in disease resistance was due to SA accumulation in the root, resulting from *EDS1*, *PAD4* and *ICS1* expression, and SA-dependent NPR1, PR1 and PR5 activation. Our findings provide insights into the molecular mechanisms in which ACS2/6-dependent ACC accumulations increase the resistance of upland cotton to *V. dahliae* infection.

## Results

### Exogenous ACC impeded *Vd991* infections of cotton plants

**Fig. 1:**
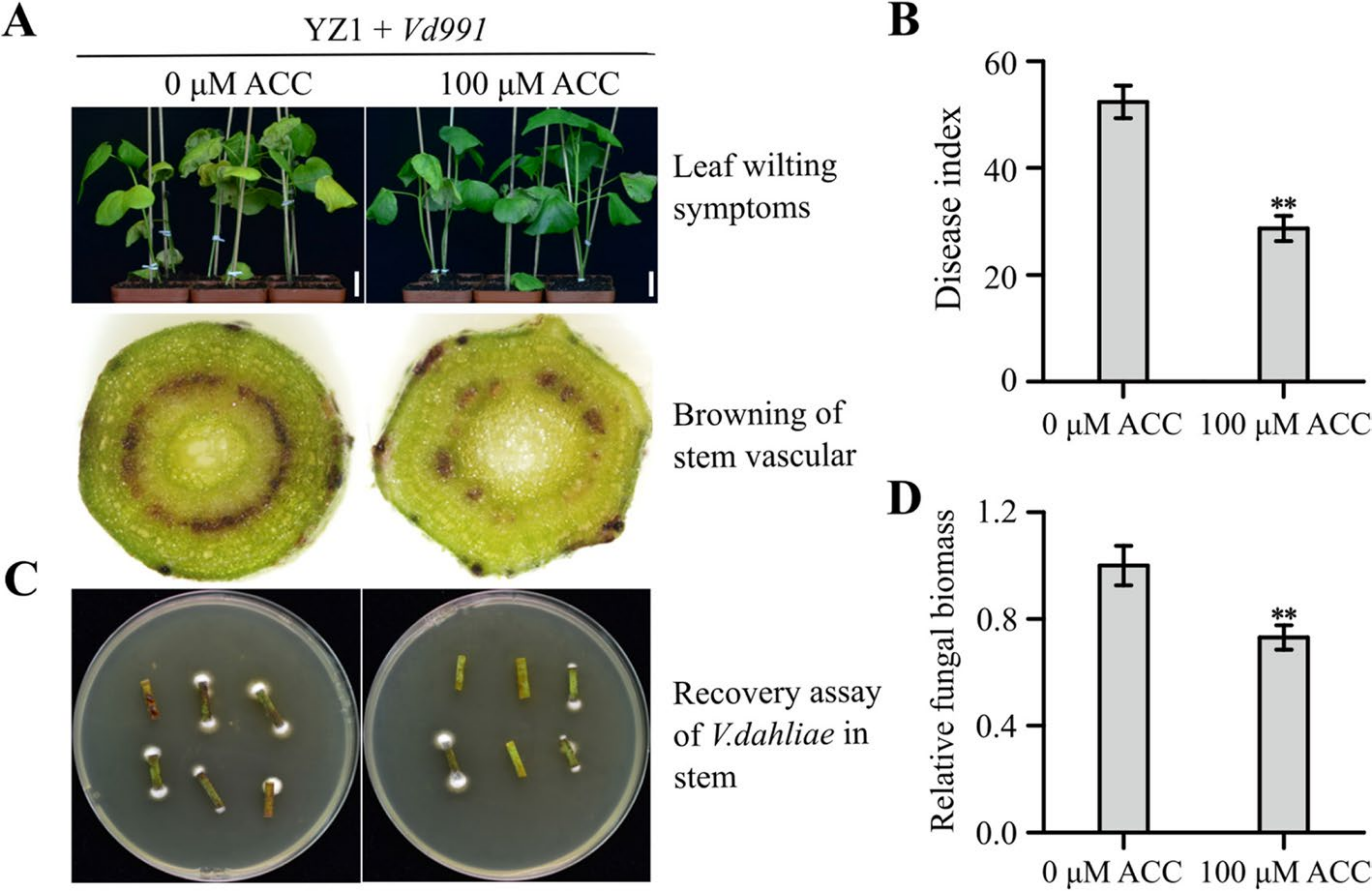
Effects of ACC treatments on cotton plants infected with *Vd991.* **A** Disease symptoms on cotton plants at 16 days after inoculation with *Vd991* with and without a 100-μM ACC treatment. Upper: leaf wilting symptoms. Bar = 2.5 cm. Bottom: stem vascular browning. **B** Disease indexes of cotton plants at 16 days after inoculation with *Vd991* with and without a 100-μM ACC treatment. Data represent means ± standard errors of three independent repeats, with at least 32 plants per replicate. **C** Recovery assay of *V. dahliae* in stems. Stem sections were collected from cotton plants at 16 days after inoculation with *Vd991* with and without a 100-μM ACC treatment and cultured on PDA medium. After 7 days, they were photographed. **D** qRT-PCR assay of *V. dahliae* biomasses in stem of cotton plants at 16 days after inoculation with *Vd991* with and without a 100-μM ACC treatment. Experiments were repeated three times with similar results. Values are means ± SD. Differences between groups were compared using Student’s *t*-test (**P < 0.01)

We first investigated whether ACC treatment affected the infection of *V. dahliae* on cotton plants. In no ACC treatment, the cotton seedlings inoculated with *Vd991* displayed the typical disease symptoms, such as leaf yellowing, wilting and vascular browning (Fig. 1A and S1A). For cotton plants pretreated with ACC (25, 50 or 100 μM), the disease symptoms caused by *Vd991* inoculation for 16 days was alleviated, for example, under the conditions of 100-μM ACC treatment, the disease index in the ACC-treated plants (28.69 ± 2.39%) was lower than that in the untreated plants (52.39 ± 3.29%) (Fig. 1B and S1B). Meantime, there was a lower fungal recovery from the stem sections collected from the inoculated plants (Fig. 1C), with the fungal biomasses in stems having decreased by 27% in the treated plants compared to the untreated plants (Fig. 1D and S1C). These data suggested that ACC was involved in the resistance response of upland cotton to *V. dahliae* infection, and the *Vd991*-associated degree of *Verticillium* wilt disease was negatively correlated with ACC concentrations.

To test whether ACC directly interfered the reproduction and growth of *Vd991* stain, we checked the growth of mycelia on PDA medium exposed to ACC (0, 50 or 100 μM). Results showed that the diameters of the growth of mycelia were no difference (Fig. S2), which suggest that ACC itself did not inhibit the fungal reproduction or growth. It is thus that ACC may act as a regulator to enhance cotton host resistance to *V. dahliae* infection.

### **Gh**ACS2/6-dependent ACC accumulation improved cotton resistance to *Vd991* infection under laboratory conditions

**Fig. 2:**
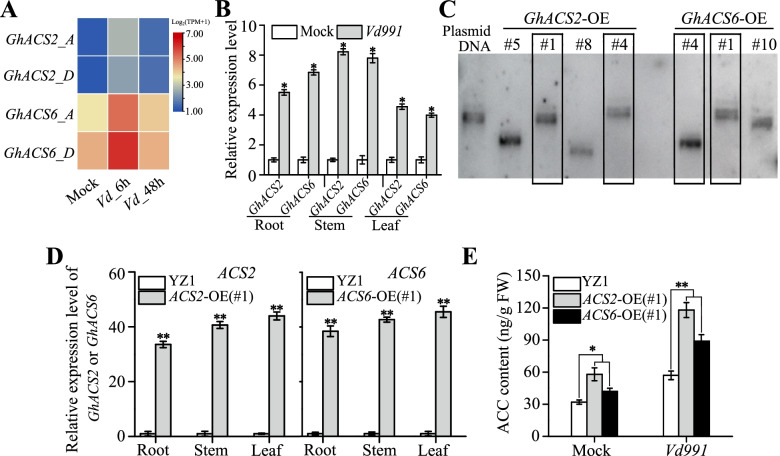
Creation of the transgenic *GhACS2-* and *GhACS6*-overexpression lines using the expression characteristics of *GhACS* genes. **A** Heatmap generated from RNA-seq data (Genome sequencing project accession: SRP118279). The relative expression levels of *GhACS2* and *GhACS6* in roots of *G. hirsutum* at 6 h and 48 h after *Vd991* inoculation. Different columns in the figure represent various samples, and rows represent genes. The color represents the level of expression of the gene (log_2_ (TPM+1)) in the sample. **B** Expression profiles of *GhACS2* and *GhACS6* in roots, stems and leaves of the *V. dahliae* susceptible YZ1 cultivars with and without *Vd991* infection as assessed by qRT-PCR. *GhUBQ7* was used as the internal control to normalize all the data. Each experiment was performed using three independent biological replicates. Differences between groups were compared using Student’s *t*-test (*P < 0.05). **C** Southern blot analysis of the expression levels of *GhACS2* and *GhACS6* in transgenic lines *GhACS2*-OE and *GhACS6*-OE, respectively. The numbers represent the transgenic lines of *GhACS2*-OE and *GhACS6*-OE. The original image was put in the supplementary figure 3. **D** qRT-PCR analysis of *GhACS2* and *GhACS6* mRNA levels in YZ1, *GhACS2*-OE and *GhACS6*-OE. *GhUBQ7* was used as the reference gene. Experiments were repeated three times with similar results. Differences between groups were compared using Student’s *t*-test (**P < 0.01). **E** HPLC analysis of ACC accumulations in roots of YZ1, *GhACS2*-OE and *GhACS6*-OE plants at 16 days after inoculation with *Vd991*. Experiments were repeated three times with similar results. Values are means ± SDs (Student’s *t*-test; *P < 0.05; **P < 0.01)

To understand the relationship between *in vivo* ACC production and *V. dahliae* infection, we investigated whether a *Vd991* infection affected the expression of *GhACS* genes in RNA-seq database (Genome sequencing project accession: SRP118279). Data analysis displayed that *Vd991* inoculation significantly increased *GhACS2* or *GhACS6* expression in roots during 48 h, especially at 6 h (Fig. 2A), which implied that the activation of *GhACS2* or *GhACS6* may be involved in early responses of cotton to *V. dahliae* infection.

To investigate this involvement, *GhACS2* and *GhACS6* expression in YZ1 seedlings in response to the pathogen inoculation was monitored using qRT-PCR. With no *V. dahliae* inoculation, *GhACS2* and *GhACS6* transcript levels were low (Fig. 2B). In response to *V. dahliae* inoculation, *GhACS2* and *GhACS6* transcripts were significantly increased in roots, stems and leaves, especially roots and stems (Fig. 2B), with the *GhACS2* expression levels in YZ1 plants being 5.5, 8.2 and 4.5 times greater, respectively, than in the control without *Vd991* inoculation (Fig. 2B), whereas the *GhACS6* expression levels in YZ1 plants were 6.8, 7.8 and 4.0 times greater, respectively, than in the control (Fig. 2B). Thus, *Vd991* inoculation induced *GhACS2* and *GhACS6* expression in cotton plants.

To determine the roles of GhACS2 and GhACS6 in cotton defenses, the *GhACS2-* and *GhACS6*-overexpression transgenic lines were created, and five *GhACS2-*OE and six *GhACS6*-OE transgenic lines with a single copy insertion were obtained (Fig. 2C, Fig. S3), respectively. Of them, the *GhACS2* and *GhACS6* expression levels was relatively high in *GhACS2-*OE(#1), *GhACS2-*OE(#4), *GhACS6*-OE(#1) and *GhACS6*-OE(#4) lines (Fig. 2D). Then, we checked the expression of the neighboring genes adjacent to *GhACS2* and *GhACS6* genes with an insertion, and results indicated that the expression of these adjacent genes did not response to *V. dahliae* infection (Fig. S4). Therefore, we selected these four transgenic lines for the following studies.

ACC content in *GhACS2-*OE(#1) and *GhACS6*-OE(#1) plants with or without *Vd991* inoculation was analyzed, and results were as follows: under no *Vd991* inoculation conditions (distilled water as the controls), after 16 days, ACC contents in roots of *GhACS2*-OE(#1) (58.1 ± 6.03 ng/g) and *GhACS6*-OE(#1) (42.6 ± 3.52 ng/g) were slightly greater than that of YZ1 (32.2 ± 2.12 ng/g). At 16 days after *Vd991* inoculation, the ACC contents in roots of *GhACS2*-OE(#1) (118.5 ± 7.32 ng/g) and *GhACS6*-OE(#1) (89.2 ± 6.24 ng/g) were significantly greater than that of YZ1 (57.1 ± 4.08 ng/g) (Fig. 2E). Similar scenario occurred in both stems and leaves (Fig. S5). Evidently, ACC accumulation was significantly increased in *GhACS2-*OE(#1) or *GhACS6*-OE(#1) plants.

Because ACC is a precursor of ethylene biosynthesis, we monitored whether ACC accumulation triggered ethylene signaling. Therefore, the expression activity of ethylene signaling genes, such as *GhEIN2*, *GhEIN3*, *GhETR1* and *GhCTR1*, were detected, and results showed that *Vd991* inoculation hardly changed the expression levels of these genes in root tissues (Fig. S6). These implied that ACC may use other pathway, instead of ethylene signaling pathway, to be involved in the resistance of cotton to *Vd991* infection.

**Fig. 3:**
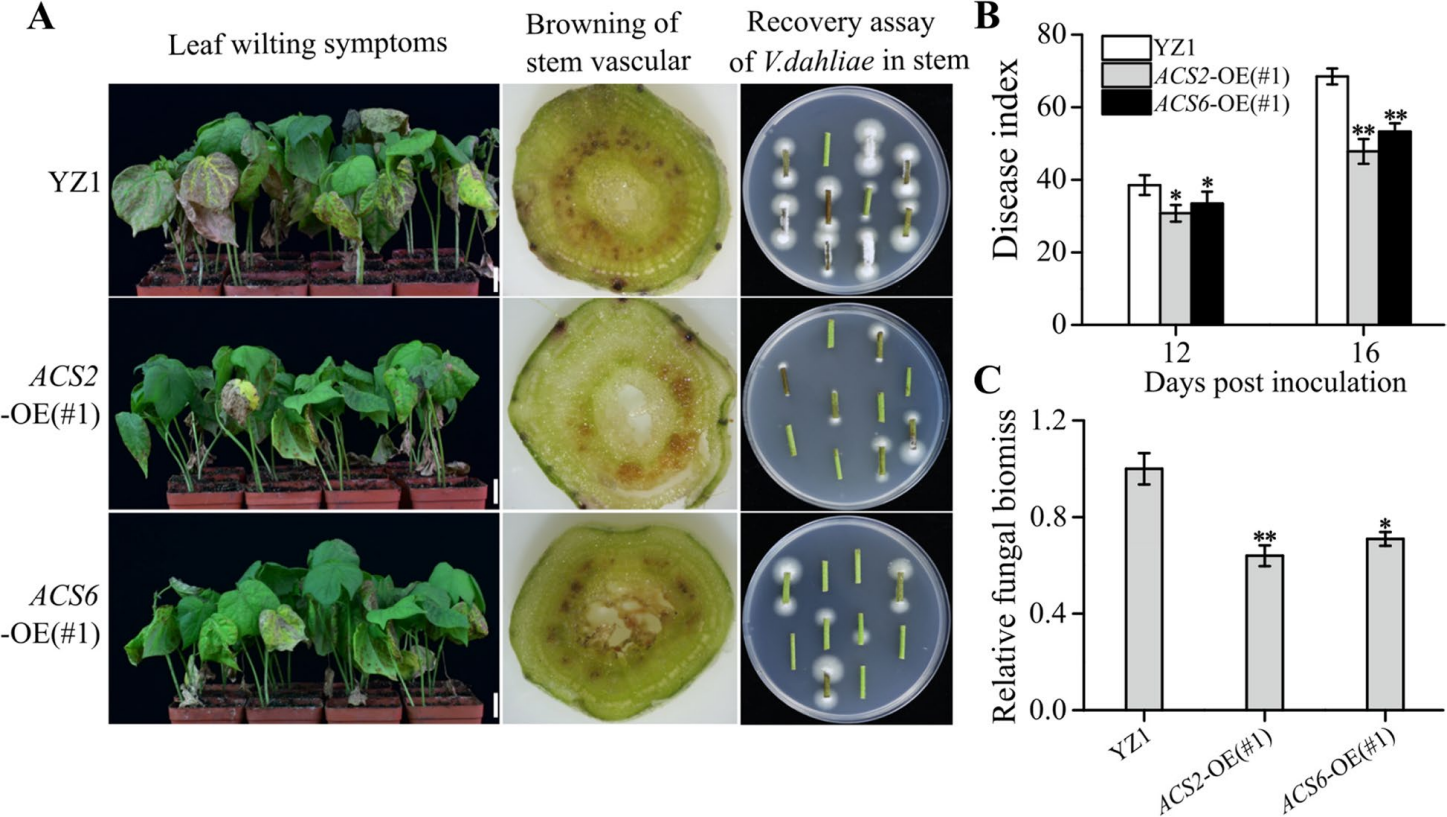
Effects of *GhACS2* and *GhACS6* overexpression on cotton plants infected with *V. dahliae* under laboratory conditions. **A** Disease symptoms, such as leaf disease symptoms (left) and stem vascular browning (middle), as well as recovery assays of *V. dahliae* in stem (right) in YZ1 (upper), *GhACS2*-OE (middle) and *GhACS6*-OE (lower) plants at 16 days after *Vd991* inoculation. Bar = 2.5 cm. **B** Disease indexes of YZ1, *GhACS2*-OE and *GhACS6*-OE plants at 12 and 16 days after inoculation with *Vd991*. (C) qRT-PCR analysis of the relative biomasses in stems of YZ1, *GhACS2*-OE and *GhACS6*-OE plants at 16 days after inoculation with *Vd991*

Verticillium wilt symptoms caused by *Vd991* infection for 16 days were investigated in ACC-accumulated plants with the mock treated YZ1 plants as the control. The survey result as follows: (1) the cotyledons of these ACC-accumulated plants exhibited less wilting and chlorotic symptoms (Fig. 3A, left panel); (2) fungal recovery assays suggested that there were more *V. dahliae* colonies in stems of YZ1 than in both *GhACS2*-OE(#1) and *GhACS6*-OE(#1) (Fig. 3A, right panel); (3) the browning of vascular tissues was more severe in YZ1 plants than in *GhACS2*-OE(#1) and *GhACS6*-OE(#1) plants (Fig. 3A, middle panel); (4) the disease indexes were significantly lower in *GhACS2*-OE(#1) (47.83 ± 3.39%) and *GhACS6*-OE(#1) (53.28 ± 2.30%) than in YZ1 (68.47 ± 2.19%) (Fig. 3B); (5) *V. dahliae* biomasses in the stems of *GhACS2*-OE(#1) and *GhACS6*-OE(#1) plants were 36% and 29% lower than in YZ1 plants (Fig. 3C). The results of *GhACS2*-OE(#4) and *GhACS6*-OE(#4) were similar to *GhACS2*-OE(#1) and *GhACS6*-OE(#1) (Fig. S7). Data suggest that *GhACS2* and *GhACS6* expression activity was needed for cotton resistance to *Vd991* infection

To further illustrate that *GhACS2* and *GhACS6* expression activity is associated with cotton *Verticillium* wilt resistance, we used VIGS system to downregulated *GhACS2* and *GhACS6* expression. In response to *Vd991* infection, the plants with silenced expression of *GhACS2* or *GhACS6* gene increased the susceptibility, while the plants silencing two genes indicated the most severe disease symptom (Fig. S8). Evidently, the expression activity of *GhACS2* and *GhACS6* genes improved cotton resistance to *Vd991* infection.

### ***Gh****ACS2/6* overexpression improved cotton resistance to *V. dahliae* infection in the artificial *Verticillium* wilt nursery

**Fig. 4:**
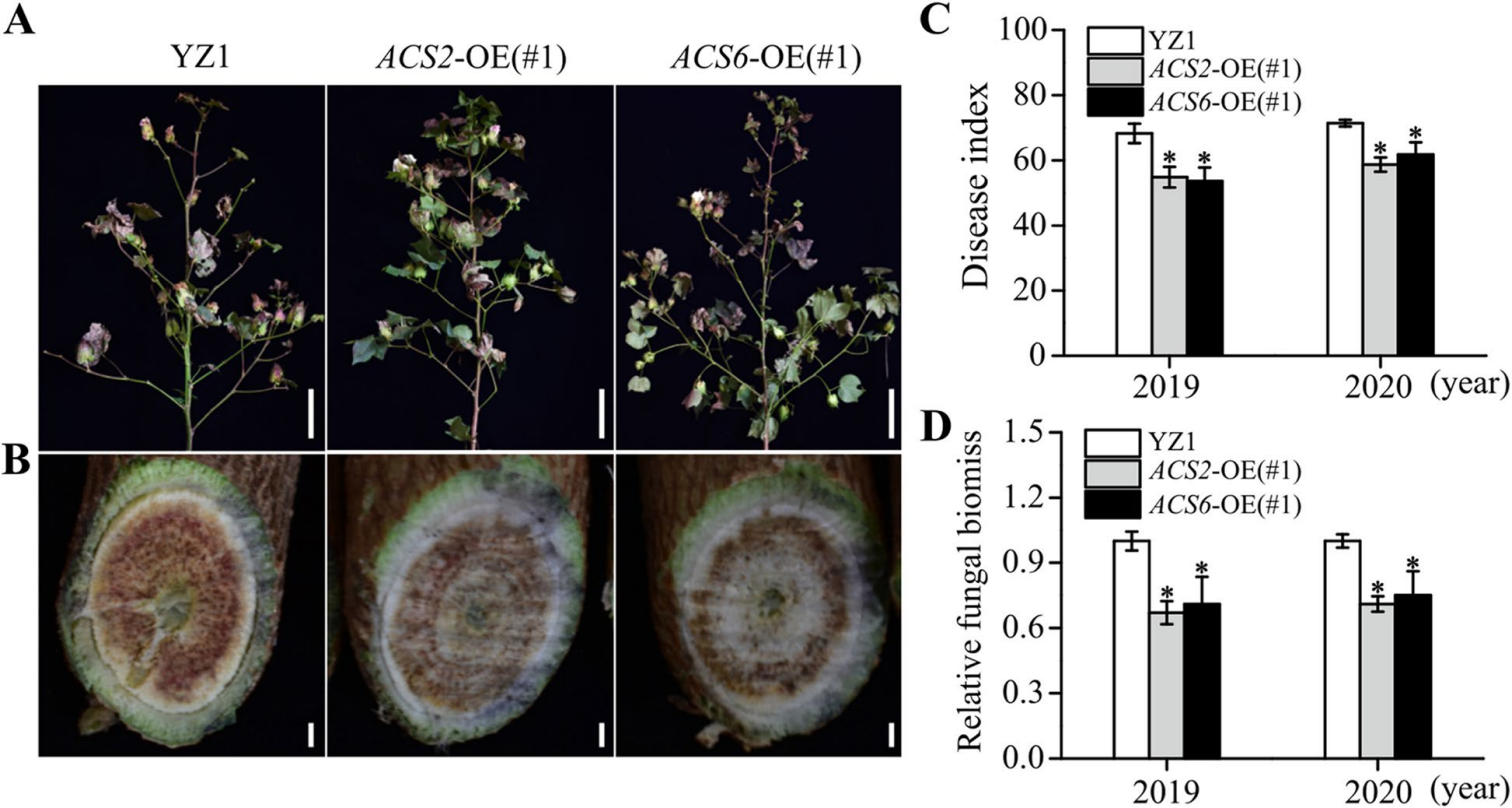
Effects of *GhACS2* and *GhACS6* overexpression on cotton plants infected with *V. dahliae* in a *Verticillium* disease nursery under open-field conditions. **A**–**C** Disease symptoms of shoots (A, bar = 20 cm) and browning of stem vascular tissues (**B**, bar = 2.5 cm), as well as the disease indexes (**C**) of YZ1, *GhACS2*-OE and *GhACS6*-OE plants growing in the disease nursery. **D** Relative *V. dahliae* biomasses in stems of YZ1, *GhACS2*-OE and *GhACS6*-OE plants growing in the disease nursery. Data were obtained using qRT-PCR, and cotton *GhUBQ7* was used as the internal control to normalize all the data. Experiments were repeated three times with similar results. Values are means ± SDs (Student’s *t*-test; *P < 0.05)

Under the strong infection conditions of open-field Verticillium wilt nursery, we further compared the resistance of *GhACS2*-OE(#1) and *GhACS6*-OE(#1) plants to *V. dahliae* with that of YZ1 in 2019 and 2020 years. Observations indicated: (1) the susceptible YZ1 plants showed typical leaf wilting (Fig. 4A) and serious vascular browning (Fig. 4B) symptoms, but *GhACS2*-OE(#1) and *GhACS6*-OE(#1) plants were alleviated (Fig. 4A and B); (2) the disease indexes of *GhACS2*-OE(#1) (2019: 54.86 ± 3.18%; 2020: 58.73 ± 2.18%) and *GhACS6*-OE(#1) (2019: 53.62 ± 4.19%; 2020: 61.73 ± 3.80%) were lower than those of YZ1 plants (2019: 68.26 ± 2.98%; 2020: 71.43 ± 1.04%) (Fig. 4C); (3) *V. dahliae* biomasses in stems also decreased in *GhACS2*-OE(#1) (decreased by 33% in 2019; decreased by 29% in 2020) and *GhACS6*-OE(#1) (decreased by 29% in 2019; decreased by 25% in 2020) compared with in YZ1 (Fig. 4D). Data suggest that the activation of GhACS2 and GhACS6 and subsequent ACC accumulation improved the resistance of cotton to *V. dahliae* infection.

### ACC treatment or ACC accumulation impeded *Vd991* colonization and **propagation** in cotton root tissues

**Fig. 5:**
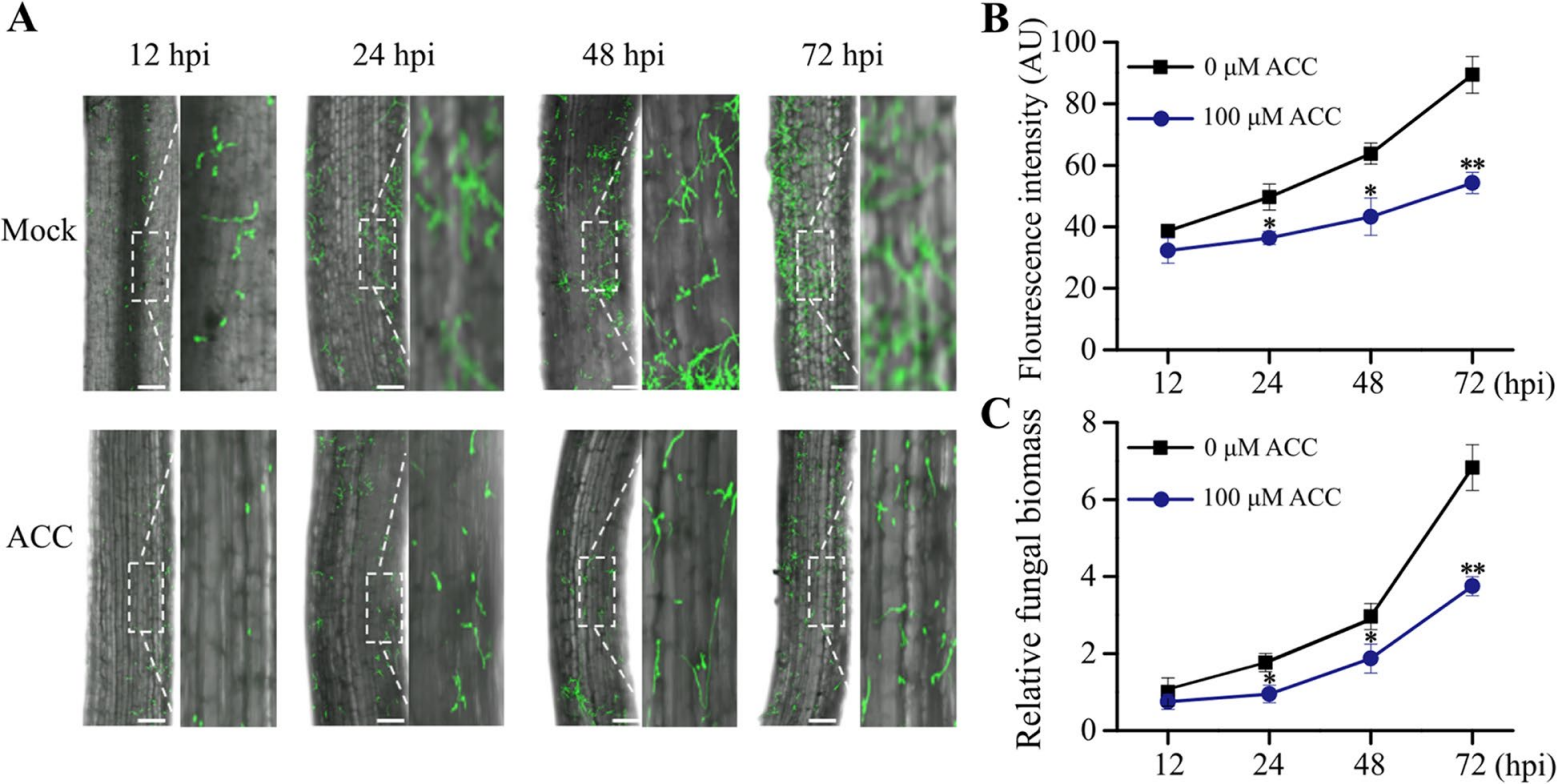
Effects of ACC treatment on GFP-tagged *Vd991* colonization process of YZ1 roots. **A** GFP fluorescence intensities of confocal scanning of YZ1 roots with or without the 100-μM ACC treatment at 12, 24, 48 and 72 h after inoculation with GFP-tagged *Vd991*. The white dotted line shows the local enlarged drawing. Bar = 20 μm. **B** Statistics of the fluorescence intensity in (A)*.* **C** Relative *V. dahliae* biomasses in the infected roots as assessed by qRT-PCR. *GhUBQ7* was used as the internal control to normalize all the data. Experiments were repeated three times with similar results. Values are means ± SDs (Student’s *t*-test; *P < 0.05, **P < 0.01)

To explain the mechanisms behind ACC’s effects on cotton resistance to *V. dahliae*, we examined whether ACC affected colonization or diffusion of *V. dahliae* in root tissues. In no ACC treatment, the GFP-marked *V. dahliae* stain showed fluorescence emissions in YZ1 roots during inoculation for 12 h. This scenario was similar to those of previous reports [14, 15]. After more time (24, 48 and 72 h), the GFP-marked *Vd991* colonization was extended and diffused. For example, the GFP fluorescence intensity at 72 h was 2.31 times greater than that at 12 h (Fig. 5A and B). However, in ACC-treated root tissues, there was less increase in GFP fluorescence intensity. For example, the GFP fluorescence intensity from ACC-treated roots was 0.67 times that from untreated roots at 72 h (Fig. 5B), which indicated that the ACC treatment reduced *V. dahliae* conidia in cotton root tissues. In addition, the fungal biomass assay indicated that the ACC treatment reduced the *V. dahliae* biomass in YZ1 root tissues. For example, *V. dahliae* inoculation for 72 h, the fungal biomass in ACC-treated root tissues was approximately 0.55 times that of the untreated root tissues (Fig. 5C). That is to say, ACC treatment hindered the *V. dahliae* invasion of cotton root tissues.

**Fig. 6:**
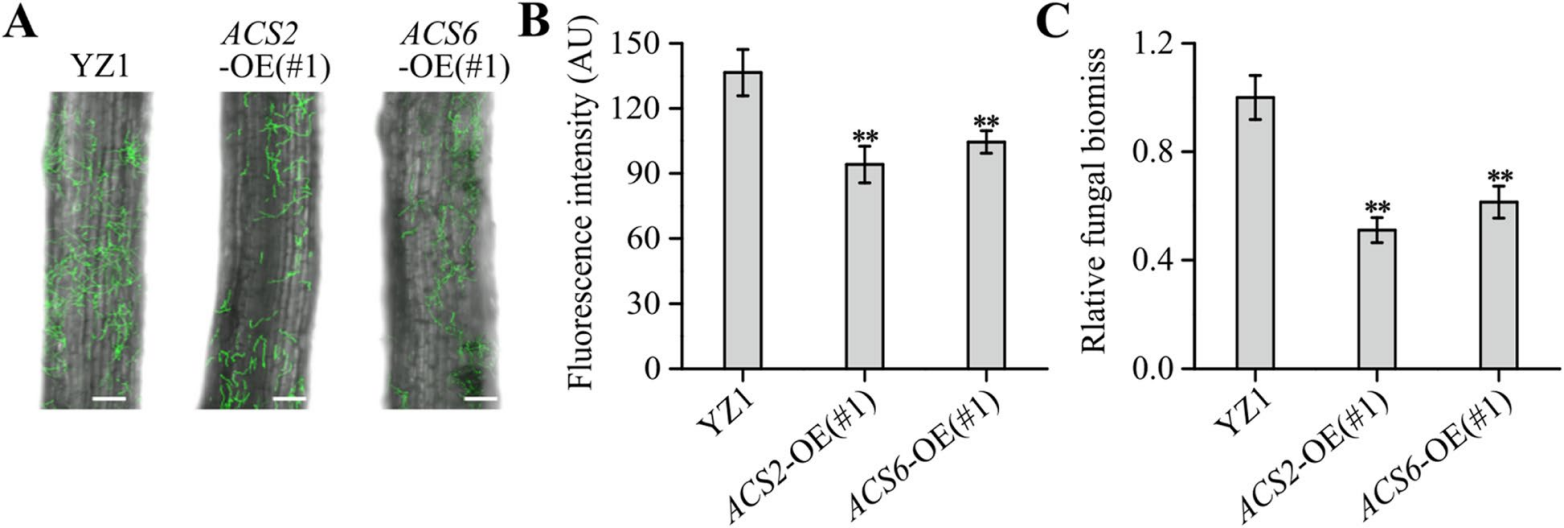
GFP-tagged *V. dahliae* colonization in roots of YZ1, *GhACS2*-OE and *GhACS6*-OE plants. **A**, **B** GFP fluorescence intensities of confocal scanning of YZ1, *GhACS2*-OE and *GhACS6*-OE roots (A, bar = 20 μm) and fluorescence intensity statistics (**B**) at 72 h after inoculation with *Vd991*-GFP. **C** qRT-PCR analysis of the relative *V. dahliae* biomasses in YZ1, *GhACS2*-OE and *GhACS6*-OE plants at 72 h after inoculation with *Vd991-*GFP. Experiments were repeated three times with similar results. Values are means ± SDs (Student’s *t*-test; **P < 0.01)

We further investigated how endogenous ACC accumulations affected *V. dahliae* colonization or reproduction in the root tissues. Confocal microscopic scanning showed that the GFP fluorescence caused by ‘*Vd991*-GFP’ inoculation was more intense in YZ1 roots than in *GhACS2*-OE(#1) and *GhACS6*-OE(#1) root tissues (Fig. 6A). A quantitative analysis showed that, *Vd991*-GFP inoculation for 72 h, the GFP fluorescence intensity in YZ1 root tissues was approximately 1.45 and 1.31 times greater than those in *GhACS2*-OE(#1) and *GhACS6*-OE(#1) root tissues, respectively, thus indicating a positive relation between increased *GhACS2/6* expression on *V. dahliae* resistance in plants (Fig. 6B). In addition, the relative fungal biomass in the YZ1 root tissues was approximately 1.96 and 1.64 times greater than those of *GhACS2*-OE(#1) and *GhACS6*-OE(#1) root tissues, respectively (Fig. 6C). These data suggested that endogenous ACC accumulations impede *V. dahliae* colonization of cotton root tissues.

### ACC increased the SA-dependent resistance of cotton to *V. dahliae* infection

It is necessary to determine how ACC affects the SA-dependent resistance of cotton to *V. dahliae* infection, because SA plays key roles in resisting fungal infections [1, 2]. Because SA production depends on the expression of *EDS1* and *PAD4* genes after *V. dahliae* infection [6], we investigated how ACC affected their expression. With no *Vd991* infection, ACC treatment increased *EDS1* and *PAD4* expression (Fig. S9A) and SA content (Fig. S9B). However, ACC significantly increased *EDS1* and *PAD4* expression (Fig. S9A) and SA production (Fig. S9B) in the root tissues infected by *Vd991* stain, compared with that in the untreated and uninfected control.

**Fig. 7:**
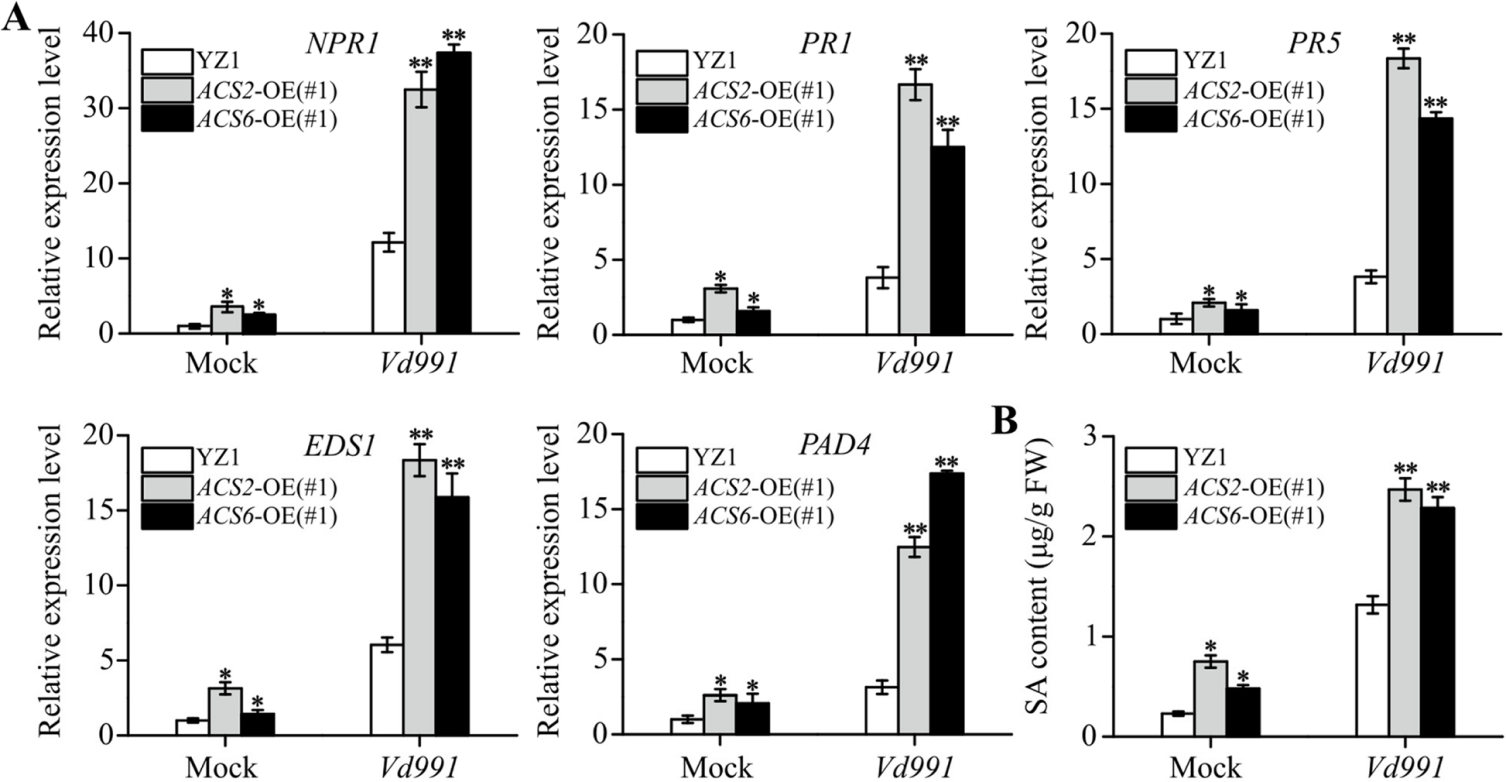
Effects of GhACS2 and GhACS6 activities on SA biosynthetic and signaling events. **A** qRT-PCR analysis of the expression levels of SA biosynthetic and signaling genes (*EDS1*, *PAD4*, *NPR1*, *PR1* and *PR5*) in *GhACS2*-OE and *GhACS6*-OE plants. *GhUBQ7* was used as the internal control to normalize all the data. Experiments were repeated three times with similar results. Values are means ± SDs (Student’s *t*-test; *P < 0.05; **P < 0.01). **B** HPLC analysis of SA levels in roots of YZ1, *GhACS2*-OE and *GhACS6*-OE plants at 16 days after *Vd991* inoculation. Experiments were repeated three times with similar results. Values are means ± SDs (Student’s *t*-test; *P < 0.05; **P < 0.01)

We further detected how ACC-accumulated plants affected SA production and signaling. With no *Vd991* infection, *EDS1* and *PAD4* expression (Fig. 7A) and SA production (Fig. 7B) were difference between the YZ1 control and the transgenic *GhACS2*-OE(#1) and *GhACS6*-OE(#1) plants. However, at 72 h after *Vd991* inoculation, *EDS1* and *PAD4* expression was significantly higher in root tissues of *GhACS2*-OE(#1) and *GhACS6*-OE(#1) than that in YZ1 (Fig. 7A). Meantime, the SA contents in *GhACS2*-OE(#1) (2.46 ± 0.11 μg/g) and *GhACS6*-OE(#1) (2.28 ± 0.11 μg/g) roots infected by *Vd991* were approximately 1.87 and 1.74 times greater, respectively, than that of YZ1 (1.31 ± 0.08 μg/g) root tissues (Fig. 7B).

Because SA induces cotton resistance to fungal infection by activating *NPR1*, *PR1* and *PR5* expression [1, 7], we further detected their gene expression levels. Without a *Vd991* infection, the expression levels of *NPR1*, *PR1* and *PR5* in roots did not significantly change when exposed to ACC treatments (Fig. S9A). However, in ACC-treated YZ1 root tissues, *NPR1*, *PR1* and *PR5* expression was significantly increased after *Vd991* infection for 72 h (Fig. S9A). Especially, the expression levels of *NPR1*, *PR1* and *PR5* were 2.68, 4.37 and 4.82 times higher in root tissues of *GhACS2*-OE(#1), respectively, and 3.08, 3.28 and 3.76 times higher in root tissues of *GhACS6*-OE(#1), respectively, than that in YZ1 root tissues (Fig. 7A). These data indicated that the increase in the ACC content of these transgenic cotton plants enhanced the SA-dependent resistance to *Vd991* infection.

## Discussion

Plants have evolved various defense mechanisms to protect themselves from invading pathogens [3]. Here, we found that cotton *ACS2-* and *ACS6-*generated ACC accumulations enhanced resistance to *V. dahliae*. Our findings lay the foundation for a comprehensive understanding of the functional evolution of ACS expression or ACC production involved plant pathogenic fungus resistance.

Cotton GhACS members play important roles in the resistance to fungal diseases. *GhACS* activity is the basis of the ACC regulation of plant responses to biotic stress [16]. Previous studies have shown that a pathogen *Pseudomonas syringae* pv. *tomato* inoculation resulted in the high expression of *ACS2* and *ACS6* genes in *Arabidopsis* [17], and *Botrytis cinerea* induced the activation of *Arabidopsis ACS2* and *ACS6*, and thus enhances the ability of *Arabidopsis* to resist infection [18]. In this study, upon *V. dahliae* inoculation, *GhACS2* and *GhACS6* genes expressions were significantly up-regulated in root, stem and leaf tissues, especially in roots and stems of the cotton cultivar YZ1 (Fig. 2, Fig.S3). These observations indicate the involvement of *ACS2* and *ACS6* expression in plant resistance to fungal diseases. Thus, we hypothesized that *ACS2* and *ACS6* overexpression may improve plant resistance to fungal diseases. For example, upon infection with the fungal pathogens *Magnaporthe oryzae* and *Rhizoctonia solani*, the rice *OsACS2-*overexpressing plants significantly increased disease resistance [19], and *GhACS2* and *GhACS6* overexpression enhanced cotton resistance to *V. dahliae* (Fig. 3, Fig. S7). Similarly, the plants with the silenced *GhACS2* and/or *GhACS6* expression enhanced the susceptibility to *Vd991* (Fig. S8). These observations suggest that activation of *GhACS2* and/or *GhACS6* was positive correlation with cotton Verticillium wilt resistance. In addition, these transgenic seedlings showed internode reducing, early maturing, and boll number slightly increasing, in comparison with that in YZ1 (Fig. 4), indicated that ACC accumulation did not harm cotton production.

ACS2*-* and ACS6*-*generated ACC accumulations induced plant resistance to *V. dahliae*. ACC applications enhance the resistance of *Arabidopsis* plants against *P. syringae* pv. *tomato* [20–22], and that ACC treatment also alleviated tomato infection by *V. dahliae* [23]. Here, observations showed that ACC treatments reduced various indicators of *V. dahliae* infection, including leaf yellowing and wilting, vascular browning, *V. dahliae* biomass, fungal recovery and disease index (Figs. 1 and 3; Fig. S1 and S7). In line with exogenous ACC treatments, endogenous ACC accumulations caused by *GhACS2* and *GhACS6* overexpression reduced these indicators of *V. dahliae* infection when cotton plants were grown in the laboratory (Figs. 1 and 3; Fig. S1) and in the *Verticillium* disease nurseries under open-field conditions (Fig. 4). It is worth noting that ACC itself did not inhibit *V. dahliae* growth and propagation (Fig. S2). These findings revealed that ACC enhanced the plant resistance to fungal pathogenicity, which may open a new avenue for through the manipulation of ACS activation or ACC accumulation. ACC may improve cotton resistance by impeding the invasion and propagation of *V. dahliae* in the root tissues. The fungus *V. dahliae* usually invades and colonizes plant roots and then spreads to shoots [3], which suggests that blocking *V. dahliae*’s colonization of roots is an important link in improving the disease resistance of cotton. Our data indicated that ACC treatments (Fig. 5) or endogenous ACC accumulations caused by *GhACS2* and *GhACS6* overexpression (Fig. 6) reduced *V. dahliae* colonization in roots.

Ethylene and SA are considered to be the classical defense of phytohormones [24]. However, the role of ACS members and ACC production in plant-fungal resistance needs clarification. Here we investigated the relationship between activation of GhACS members and SA production and SA signaling during cotton infection with *V. dahliae*. SA biosynthesis plays a key role in resistance to fungal infection [1, 2], and SA production depends on the expression of *EDS1* and *PAD4* genes after *V. dahliae* infection [6]. Assays of the effects of ACC on SA production showed that ACC treatments (Fig. S9) or endogenous ACC accumulations caused by *GhACS2* and *GhACS6* overexpression (Fig. 2E) increased the *EDS1* and *PAD4* expression levels, as well as the SA content (Fig. 7). These findings were consistent with the previous studies, namely increases in the SA contents of pathogen-challenged plant tissues result in the induction of *PR* genes and enhance resistance to a broad range of pathogens [24]. Our observations showed that ACC treatments or endogenous ACC accumulations caused by *GhACS2* and *GhACS6* overexpression (Fig. 2E) enhanced *NPR1*, *PR1* and *PR5* expression in response to *V. dahliae* infection (Fig. 7; Fig. S9). These results were consistent with a previous report in which *V. dahliae* infection enhances the expression of the SA-induced genes *PR1* or *PR5* in cotton plants [25]. These suggest that ACC enhanced SA-dependent cotton host resistance to *V. dahliae infection*. Furthermore, *V. dahliae* inoculation hardly changed the expression activity of ethylene receptor genes (*GhEIN2*, *GhEIN3*, *GhETR1* and *GhCTR1*) in root tissues (Fig. S6). Therefore, we speculate that, in the early stage of cotton young root infection with *V. dahliae*, ACC produced by cotton ACS2 and ACS6 acts as a signaling molecule in SA signaling pathway, instead of the ethylene signaling pathway.

In brief, GhACS2/6-generated ACC accumulation enhanced resistance to *V. dahliae* in a SA-dependent manner in upland cotton. Our work provides a theoretical basis for better understanding the molecular genetic mechanisms of ACC-dependent resistance to *V. dahliae*.

### Conclusion

Here we observed the effects of exogenous ACC treatments and endogenous ACC accumulations caused by *GhACS2* and *GhACS6* overexpression on the disease incidence and disease index of cotton plants, and results indicated that ACC enhanced the resistance of cotton by impeding *V. dahliae* colonization of cotton roots in an SA-dependent manner. Our research provides new insights into *GhACS2*- and *GhACS6*-mediated ACC accumulations enhanced the resistance of cotton to *V. dahliae* and new candidate for introducing resistance to Verticillium wilt in affected crops.

## Methods

### Cotton plants and growth conditions

Seeds of upland cotton YZ1 were provided Dr Gao (State Key Laboratory of Cotton Biology, Henan Province, China) [25]. In the greenhouse, the growth conditions of cotton seedlings were 25 ± 2°C, 80% relative humidity, 120 μmol m^−2^ s^−1^ light intensity and a 16-h light/8-h dark photoperiod. A field experiment of Verticillium disease nurseries were conducted at the Institute of Cotton Research of Chinese Academy of Sciences (Anyang, Henan Province, China), which has been tested for about 50 microsclerotia per gram of soil. Each block was 5 m long with two rows (0.8 m between two rows). Seeds were sown with a within-row plant-to-plant distance of 25 to 30 cm. The experiments were repeated two years of 2019 and 2020 with 50 plants per replicate.

### Creation of transgenic cotton materials and southern blot

The transgenic *GhACS2/6*-OE lines were created as previously reported [25, 26]. Briefly, the open reading frame of *GhACS2* or *GhACS6* was inserted into the vector pK7WG2 with CaMV 35S promoter, respectively, and introduced into *Agrobacterium tumefaciens* strain EHA105, and then used to infect the hypocotyl of YZ1 seedings, respectively. The positive plants were screened on 1/2 MS medium containing 50 μg/mL kanamycin, until T_3_ lines for research analysis were obtained.

Genomic DNA was extracted from young leaves of YZ1 using a plant genomic DNA kit (TIANGEN Biotech, Beijing, China). For Southern blotting, 20 μg of genomic DNA was digested with the restriction enzyme *Hind*III overnight, separated on a 0.8% agarose gels by electrophoresis and transferred onto a positively charged nylon membrane (Millipore, Billerica, MA, USA). The nylon membrane was hybridized with DIG-11-dUTP-labeled fragments at 45°C. A DIG High Primer DNA Labeling and Detection Starter kit II (Roche, Basel, Switzerland) was used for labeling and hybridization in accordance with the manufacturer’s protocol. Homozygous seedlings from single insertions and high expression lines were selected for the following studies.

### Preparation of *Vd991* and ‘*Vd991*-GFP’s spore suspensions

Because *V. dahliae* strain *Vd991* is highly virulent on its original host *G. hirsutum* [27], *Vd991* strain was used in this work. ‘*Vd991*-GFP’ is a GFP-marked *Vd991* strain [28]. Either *Vd991* or ‘*Vd991*-GFP’ strains was cultured in PDA medium at 25°C for 5 days. High activity hyphae were transferred into Czapek liquid medium and cultured for 4 days at 25°C and 120 rpm to produce conidia. Conidia were obtained by centrifugation (150 rpm for 10 min) and counted using a hemocytometer. The inoculation concentrations were adjusted as necessary.

### *V. dahliae* strain inoculations and ACC treatments

Inoculations were performed by dipping the intact roots of 3-week-old cotton seedlings in a suspension of *V. dahliae* spores (1 × 10^7^ conidia/mL) for 1 min as previously described [29], following which the seedlings were replanted into potting soil. Uninoculated plants were dipped in sterile distilled water as the controls. Refer to the ACC-treatment methods provided in the literature to study the effects of ACC on disease development [23], root drenching cotton plants with different concentrations of ACC solution 12 h prior to *Vd991* inoculation

### Analysis on the disease index

According to the previous description [30], the diseased plants were counted and divided into five levels based on their disease severity. The disease index was calculated with the following formula: disease index = [(∑disease grades × number of infected plants) / (total checked plants × 4)] × 100. All of the experiments were repeated at least three times with 32 plants per replicate.

### Quantification of *V. dahliae* biomass

Quantification of *V. dahliae* biomass was performed in accordance with previously described methods [31]. The 1-cm stem sections above the cotyledon node were ground to a powder, and an aliquot of approximately 100 mg was used for DNA isolation [32]. Quantitative real-time PCR was conducted using a Roche 480 real-time PCR system (Roche, Basle, Sweden). To measure the *V. dahliae* biomass, the internal transcribed spacer region of the ribosomal DNA was targeted using the fungus-specific ITS1-F primer [33] in combination with the *V. dahliae*-specific reverse primer ST-VE1-R [34], generating a 200-bp amplicon. The average fungal biomass was determined using at least five *Vd991*-inoculated plants for each line. The relative fold changes of the target genes were calculated as described [35]. The reference gene was the cotton ubiquitin 7 (*UBQ7*) gene. The primers used for PCR amplification are listed in Table S1.

### Recovery assay of *V. dahliae* and browning of stem vascular

1-cm stem sections above the cotyledon node were collected from cotton seedlings with *Vd991* inoculation for 16 days, surface sterilized with 0.1% HgCl_2_ for 5 min and cleaned up. Stem sections were incubated on PDA medium (at 25°C for 7 days) and photographed using a digital camera (Canon 760D, Tokyo, Japan). To observe the browning of vascular bundles in stems, stem sections above the cotyledon node were taken from cotton seedlings with *Vd991* inoculation for 16 days, and photographed using a stereo microscope (Olympus, Tokyo, Japan).

### RNA analysis

Total RNA was extracted from cotton using an RNAprep Pure Plant kit (TIANGEN Biotech). First-strand complementary DNA (cDNA) was synthesized using a Reverse Transcription system (Toyobo, Osaka, Japan) and was used as the template for qRT-PCR analyses along with 2× SYBR Green I master mix (Vazyme, Nanjing, China). qRT-PCR analyses were performed on a Roche 480 real-time PCR system (Roche, Basle, Sweden). The relative fold changes of the target genes were calculated as described [35]. The reference gene was cotton *UBQ7.* The primers used for PCR amplification are listed in Table S1.

Transcriptome data from *G. hirsutum* roots inoculated with *V. dahliae* (Genome sequencing project accession: SRP118279) were downloaded from the NCBI Sequence Read Archive database. A standard analysis of the raw expression data from the transcriptome was performed [36–38]. Log_2_(TPM + 1) normalization was performed on the expression data. The standardized data were compiled using the R-4.0.2 language.

### Confocal microscopic scanning of *V. dahliae* colonization in roots

A laser scanning confocal microscope was used to monitor GFP-marked *Vd991* colonization. Clean roots from cotton plants were inoculated with *V. dahliae* for 12, 24, 48 or 72 h as previously reported [14]. The elongation zones of roots were sectioned longitudinally by hand using a razor blade into 0.1- to 0.2-mm-thick slices. The GFP fluorescence intensities from root samples were recorded using a Nikon A1 Plus laser scanning confocal microscope (Nikon, Tokyo, Japan) with the scanning parameters of 488-nm excitation and 500–550-nm emission. Fifteen replicates of each line were included in each assay and three independent assays were performed.

### Measurements of ACC or SA contents

Measurement of endogenous ACC or SA were performed as described [39]. Fresh root, stem or leaf samples were harvested and ground into a powder, respectively. Three replicates of each frozen sample (approximately 100 mg per replicate) were ground to a fine powder in liquid nitrogen and were mixed with 750 μl of cold extraction buffer (80:19:1 methanol: water: acetic acid, vol/vol/vol). After shaking for 16 h at 4°C in the dark, the supernatants were collected. Filtrates were dried using nitrogen gas at room temperature and were then dissolved in 200 μl of methanol. For quantification, an aliquot of dissolved sample was further diluted 100 times. Supernatants were analyzed using an Applied Biosystems MDS SCIEX 4000 QTRAP liquid chromatography-tandem mass spectrometry system (AB Sciex, Foster City, CA, USA). ACC and SA standards (Sigma-Aldrich, Steinheim, Germany) were used for the quantitative analyses.

### Statistical analysis

All of the experiments were independently repeated using at least three biological replicates and three technical replicates. Data are presented as means ± SDs. Statistical significance was determined using Student’s *t*-tests. *P*-values < 0.05 (*) indicate significant differences, and *P*-values < 0.01 (**) indicate extremely significant differences.

## Supplementary Information

**Additional file 1:** **Table S1.**

**Additional file 2:** **Figures.**

## Data Availability

Data generated or analyzed during this study are included in this article and its supplemental files. Sequence data for the genes described in this study were downloaded from the Cotton Functional Genomics (https://www.cottongen.org/) or NCBI (https://www.ncbi.nlm.nih.gov/genbank/) websites. The RNA-Seq data from G. hirsutum roots inoculated with *V. dahliae* (Genome sequencing project accession: SRP118279) were downloaded from the NCBI Sequence Read Archive database and analyzed using TBtools sofeware. Accession numbers of genes are: *GhACS2* (Gh_D11G0974; *GhACS2_D*), *GhACS6* (Gh_A12G2673; *GhACS6_A*) and *UBQ7* (DQ116441).

## Abbreviations

*G. hirsutum*: *Gossypium hirsutum* L.
*V. dahliae*: *Verticillium dahliae*
ACC: 1-aminocyclopropane-1-carboxylic acid
ACS: ACC synthase
SA: Salicylic acid
GFP: Green fluorescent protein
qRT-PCR: Quantitative reverse transcription polymerase chain reaction
CaMV: Cauliflower mosaic virus
MS: Murashige and Skoog
*EDS1*: Enhanced Disease Susceptibility 1
*PAD4*: Phytoalexin-deficient 4
*ICS1*: Isochorismate synthase 1
*NPR1*: Pathogenesis-related protein 1
*PRs*: Pathogenesis-related proteins
h: Hour
