# Supplementary material for: Effect of 1-aminocyclopropane-1-carboxylic acid accumulation on Verticillium dahliae infection of upland cotton
Source: BMC Plant Biol. 2022 Aug 3;22:386. doi: 10.1186/s12870-022-03774-8 (PMC9347136; doi:10.1186/s12870-022-03774-8)
Supplement: Supplementary file 1 — Additional file 1: Table S1. [file 12870_2022_3774_MOESM1_ESM.docx]

**Table S1** Primers used in this study

| *ITS1*-F | AAAGTTTTAATGGTTCGCTAAGA |  |
| --- | --- | --- |
| *STVe1*-R | CTTGGTCATTTAGAGGAAGTAA |  |
| *GhACS2*-F | ATAGTTATGAGCGGCGGAGC |  |
| *GhACS2*-R | GTACGCCACCTTAGATCCCG |  |
| *GhACS6*-F | GTGAGCTGTGCTCGGAAAA | |
| *GhACS6*-R | TCTTTGCTCTCCACAATGAATCTA | |
| *GhEDS1*-F | CGGCGGCACCAACAAGATTATG | |
| *GhEDS1*-R | GCTTCCACTCTCGTCGAGTGAC | |
| *GhPAD4*-F | CCCTAGTGCTCAAGCCAAGGTC | |
| *GhPAD4*-R | GGATGGAAGAATGGAAAGAAATGAA | |
| *GhNPR1*-F | GAACTAGGAAAGCAGACTAAGGAACCA | |
| *GhNPR1*-R | GCGAATCGGCTTTCTTCTTCA | |
| *GhPR1*-F | CACGTGGTGCTGTTGTTGTTACTG | |
| *GhPR1*-R | AAGAATGTGGGTTAGTGAGAGGGT | |
| *GhPR5*-F | ACCACTTGAGTATAATGCCCGC | |
| *GhPR5*-R | GCCGTGATTCATACAGTTATCCTCA | |
| *GhUB7*-F | GAAGGCATTCCACCTGACCAAC | |
| *GhUB7*-R | CTTGACCTTCTTCTTCTTGTGCTTG | |
