# Supplementary material for: Effect of 1-aminocyclopropane-1-carboxylic acid accumulation on Verticillium dahliae infection of upland cotton
Source: BMC Plant Biol. 2022 Aug 3;22:386. doi: 10.1186/s12870-022-03774-8 (PMC9347136; doi:10.1186/s12870-022-03774-8)
Supplement: Supplementary file 2 — Additional file 2: Figures. [file 12870_2022_3774_MOESM2_ESM.zip › Original Figures.docx]

**Figure 1A**


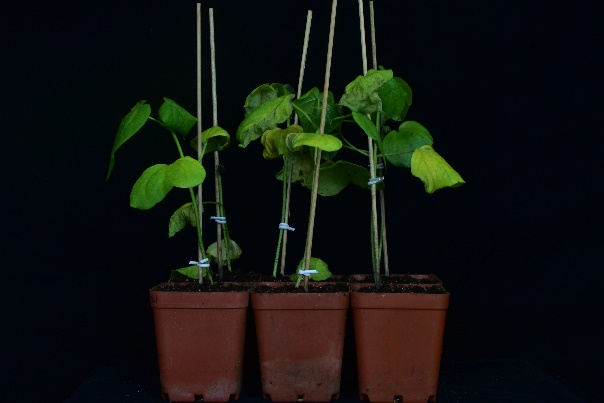

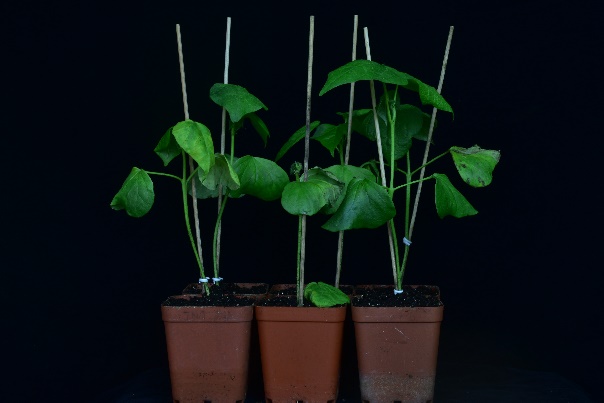


Fig1A 0µM ACC_*Vd991* Fig1A 100µM ACC_*Vd991*


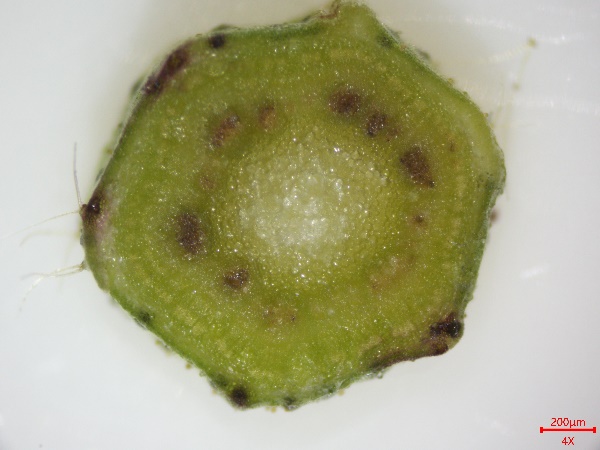

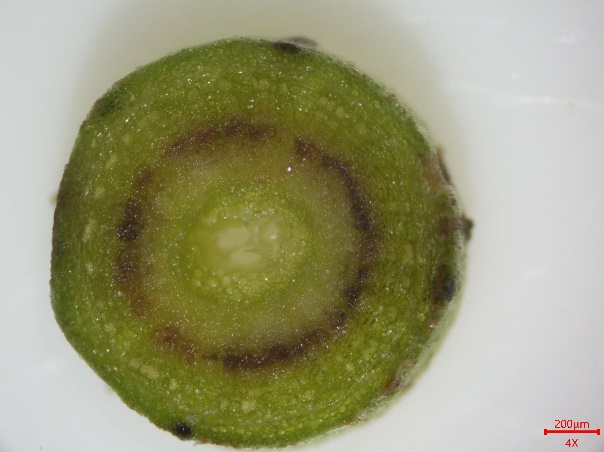


Fig1A 0µM ACC_*Vd991* stem vascular Fig1A 100µM ACC_*Vd991* stem vascular


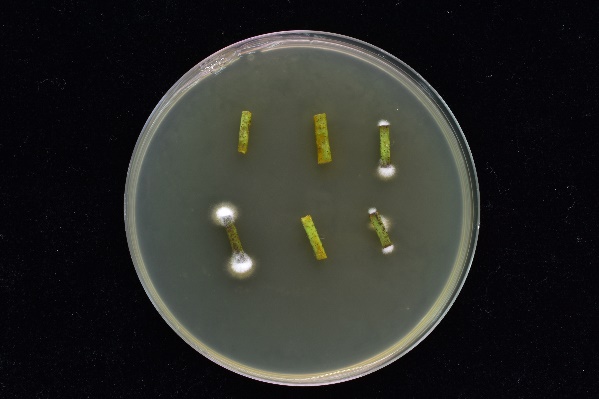

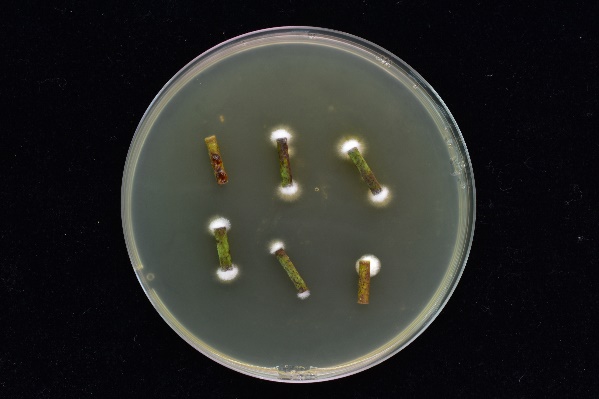


Fig1C 0µM ACC_*Vd991* Recovery assay Fig1C 100µM ACC_*Vd991* Recovery assay

**Figure 2C**


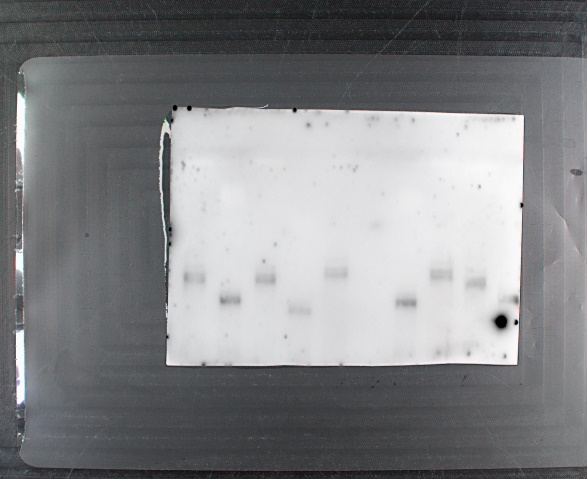


**Figure 3A**


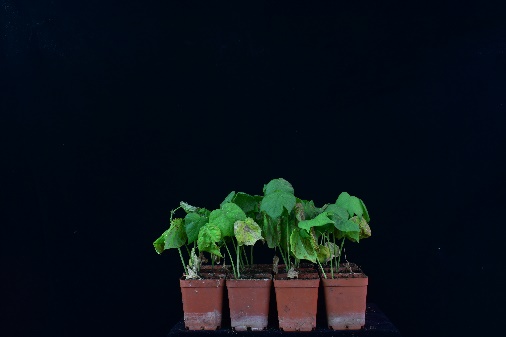

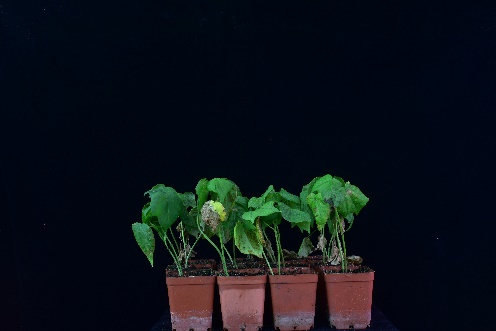

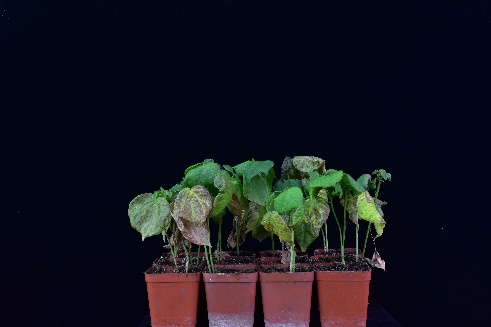


YZ1 *ACS2*-OE(#1) *ACS6*-OE(#1)


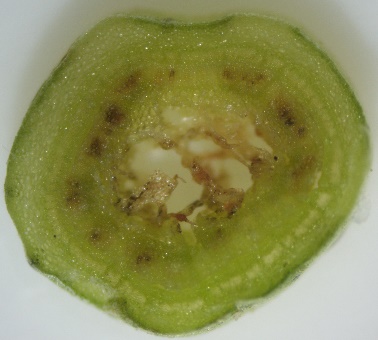

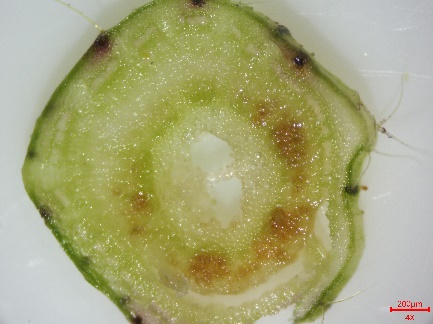

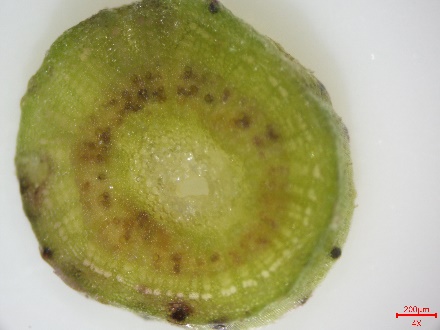


YZ1 stem vascular *ACS2*-OE(#1) stem vascular *ACS6*-OE(#1)recovery assay


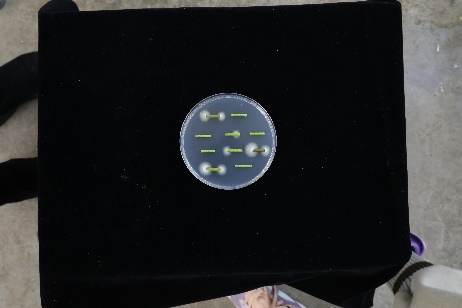

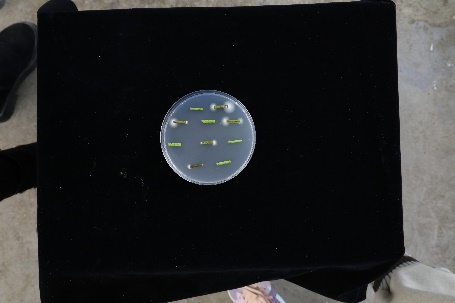

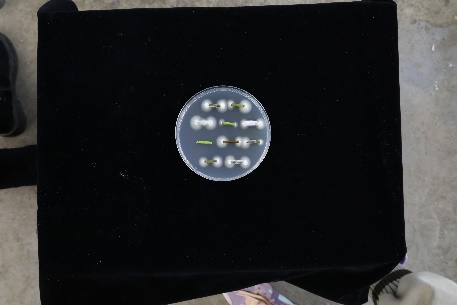


YZ1 recovery assay *ACS2*-OE(#1)recovery assay *ACS6*-OE(#1)recovery assay

**Figure 4A**


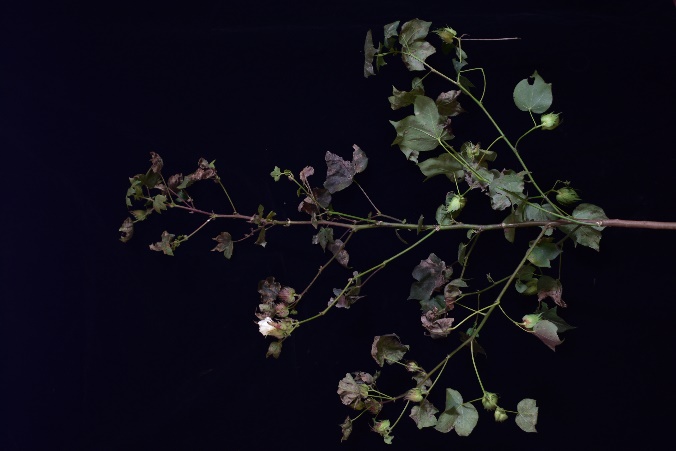

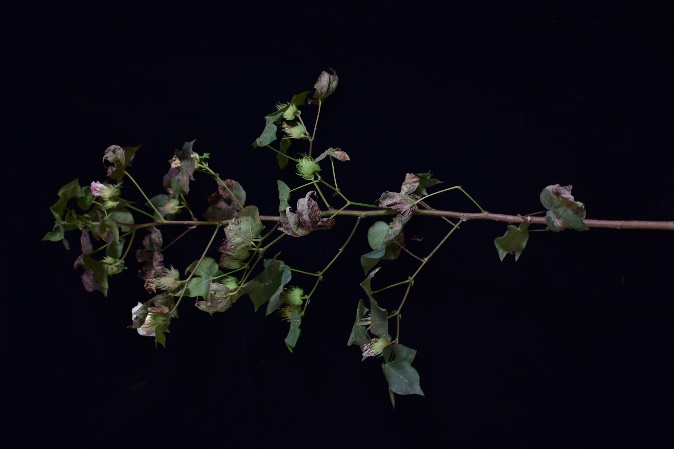

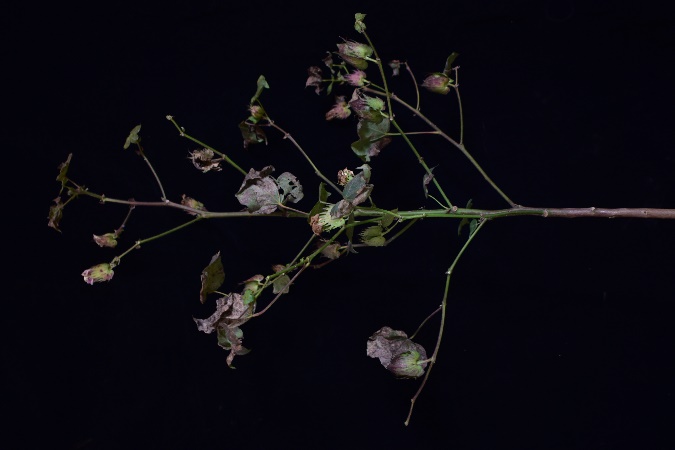


YZ1  *ACS2*-OE(#1) *ACS6*-OE(#1)


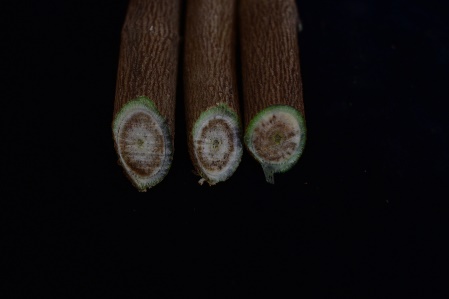

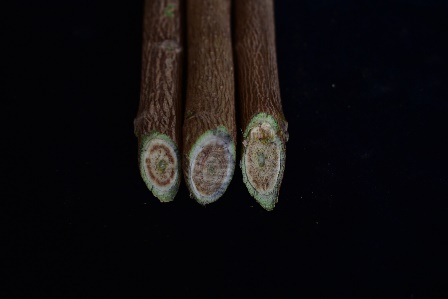

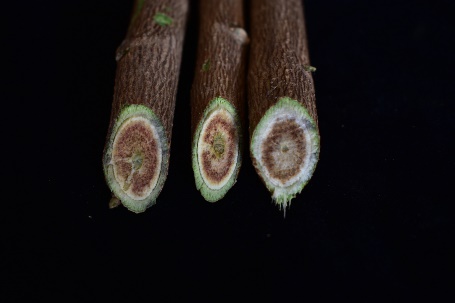


YZ1  *ACS2*-OE(#1) *ACS6*-OE(#1)

**Figure 5A**


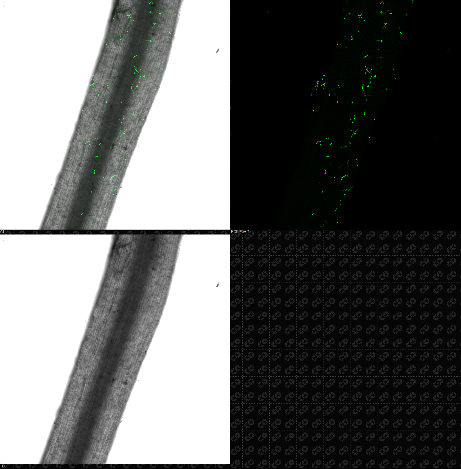

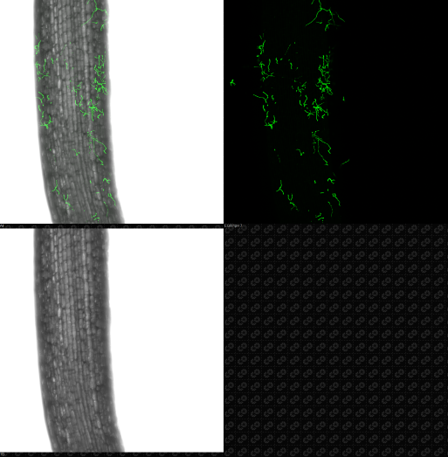


Mock-12h Mock-24h


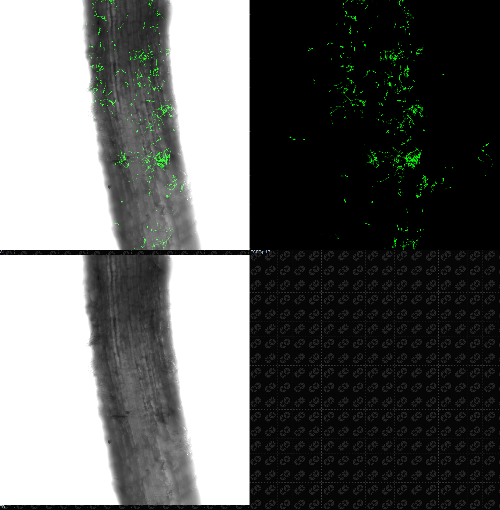

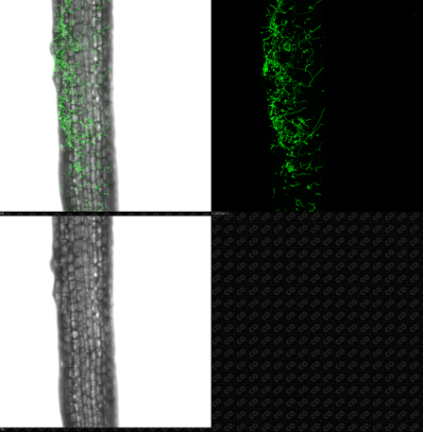


Mock-48h Mock-72h


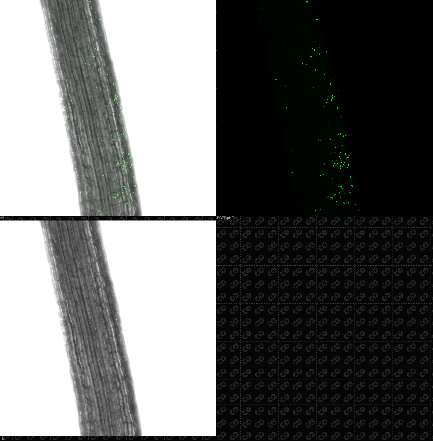

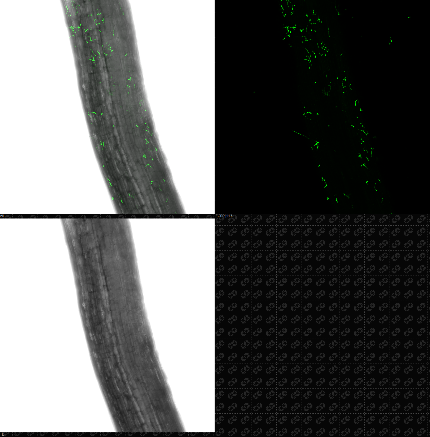


100µM ACC-12h 100µM ACC-24h


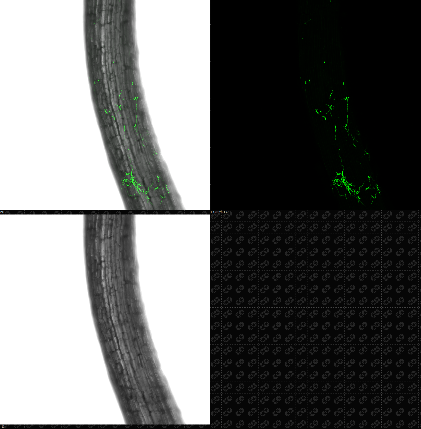

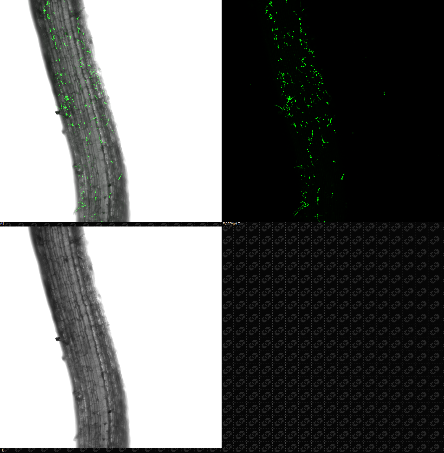


100µM ACC-48h 100µM ACC-72h

**Figure 6A**


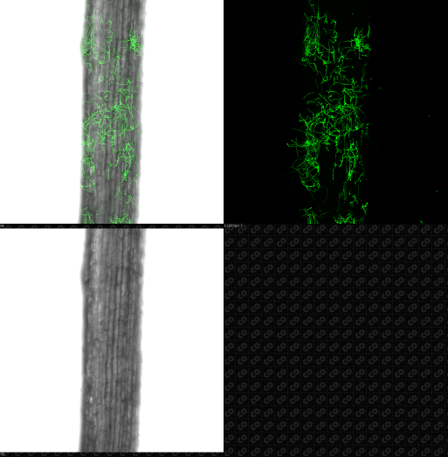

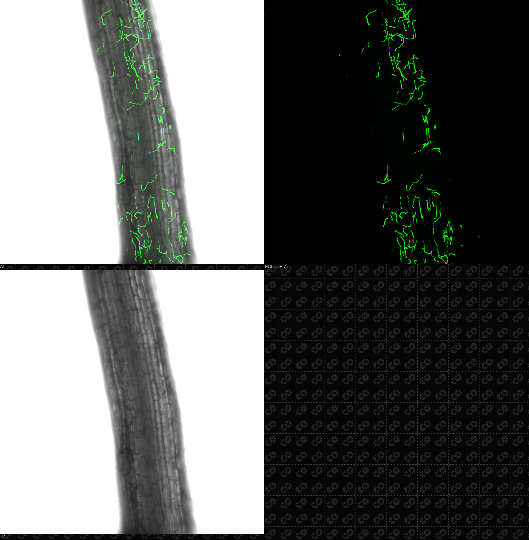


YZ1-72h  *ACS2*-OE(#1)_72h


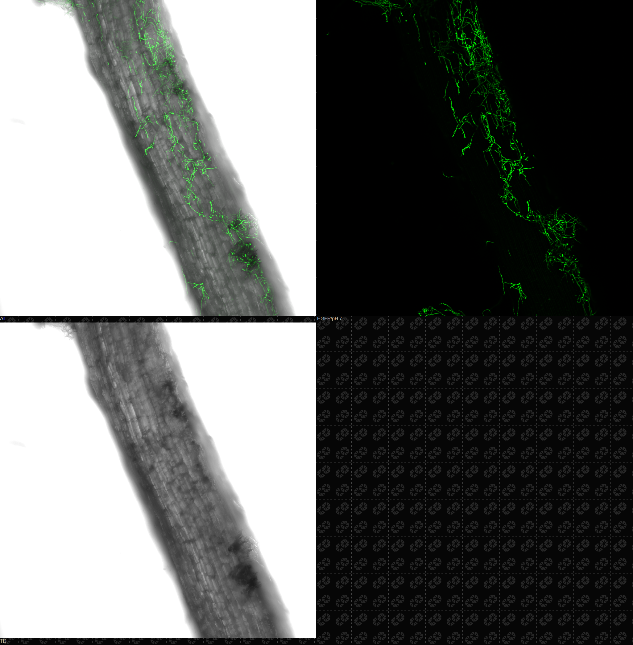


*ACS6*-OE(#1)_72h
