# Supplementary material for: Effect of 1-aminocyclopropane-1-carboxylic acid accumulation on Verticillium dahliae infection of upland cotton
Source: BMC Plant Biol. 2022 Aug 3;22:386. doi: 10.1186/s12870-022-03774-8 (PMC9347136; doi:10.1186/s12870-022-03774-8)
Supplement: Supplementary file 2 — Additional file 2: Figures. [file 12870_2022_3774_MOESM2_ESM.zip › Supplementary Figures re-submit.pptx]

## Slide 1
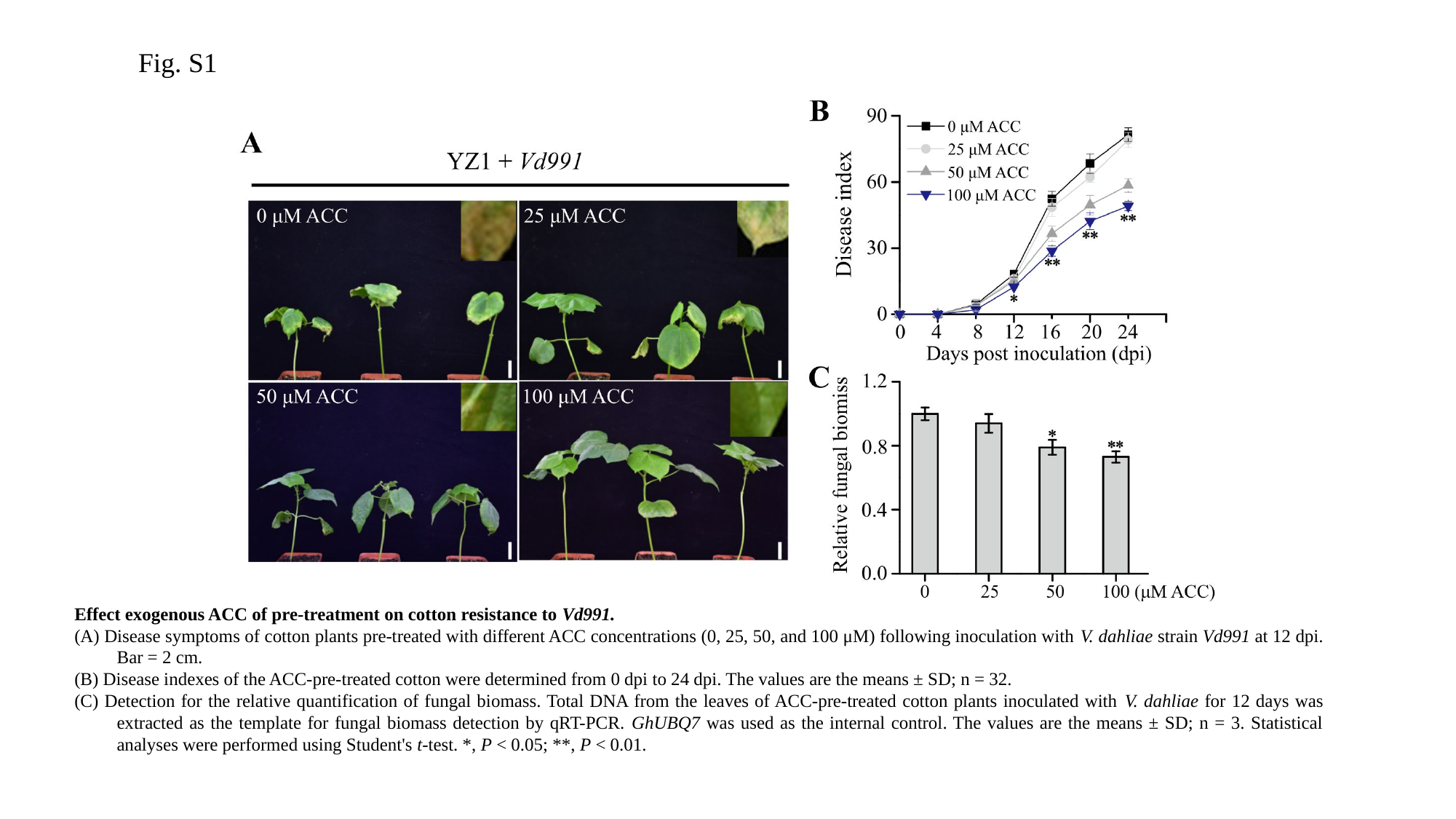

Fig. S1
Effect exogenous ACC of pre‐treatment on cotton resistance to Vd991.
(A) Disease symptoms of cotton plants pre‐treated with different ACC concentrations (0, 25, 50, and 100 μM) following inoculation with V. dahliae strain Vd991 at 12 dpi. Bar = 2 cm.
(B) Disease indexes of the ACC‐pre‐treated cotton were determined from 0 dpi to 24 dpi. The values are the means ± SD; n = 32.
(C) Detection for the relative quantification of fungal biomass. Total DNA from the leaves of ACC‐pre‐treated cotton plants inoculated with V. dahliae for 12 days was extracted as the template for fungal biomass detection by qRT-PCR. GhUBQ7 was used as the internal control. The values are the means ± SD; n = 3. Statistical analyses were performed using Student's t-test. *, P < 0.05; **, P < 0.01.

## Slide 2
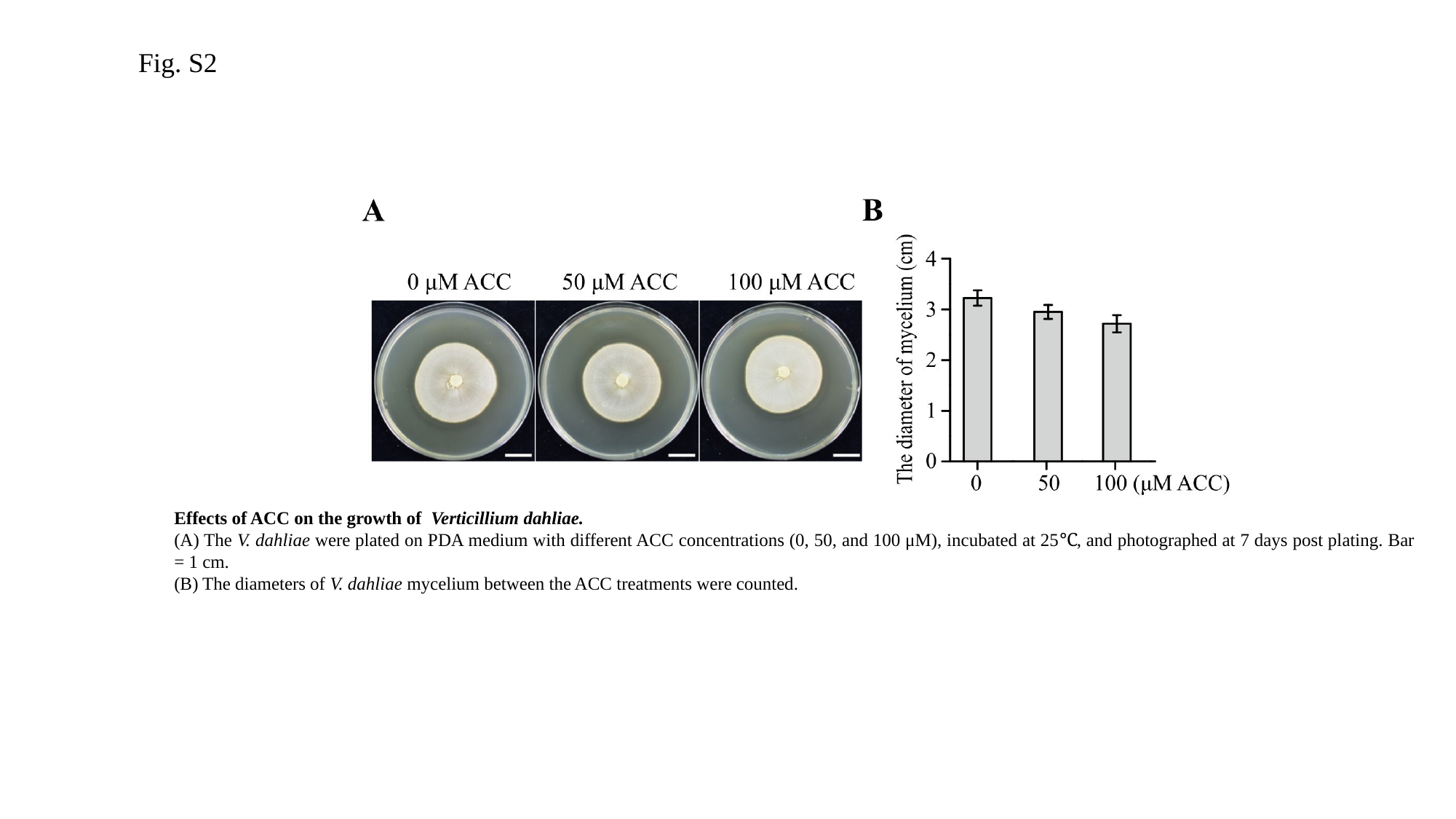

Fig. S2
Effects of ACC on the growth of  Verticillium dahliae.
(A) The V. dahliae were plated on PDA medium with different ACC concentrations (0, 50, and 100 μM), incubated at 25℃, and photographed at 7 days post plating. Bar = 1 cm.
(B) The diameters of V. dahliae mycelium between the ACC treatments were counted.

## Slide 3
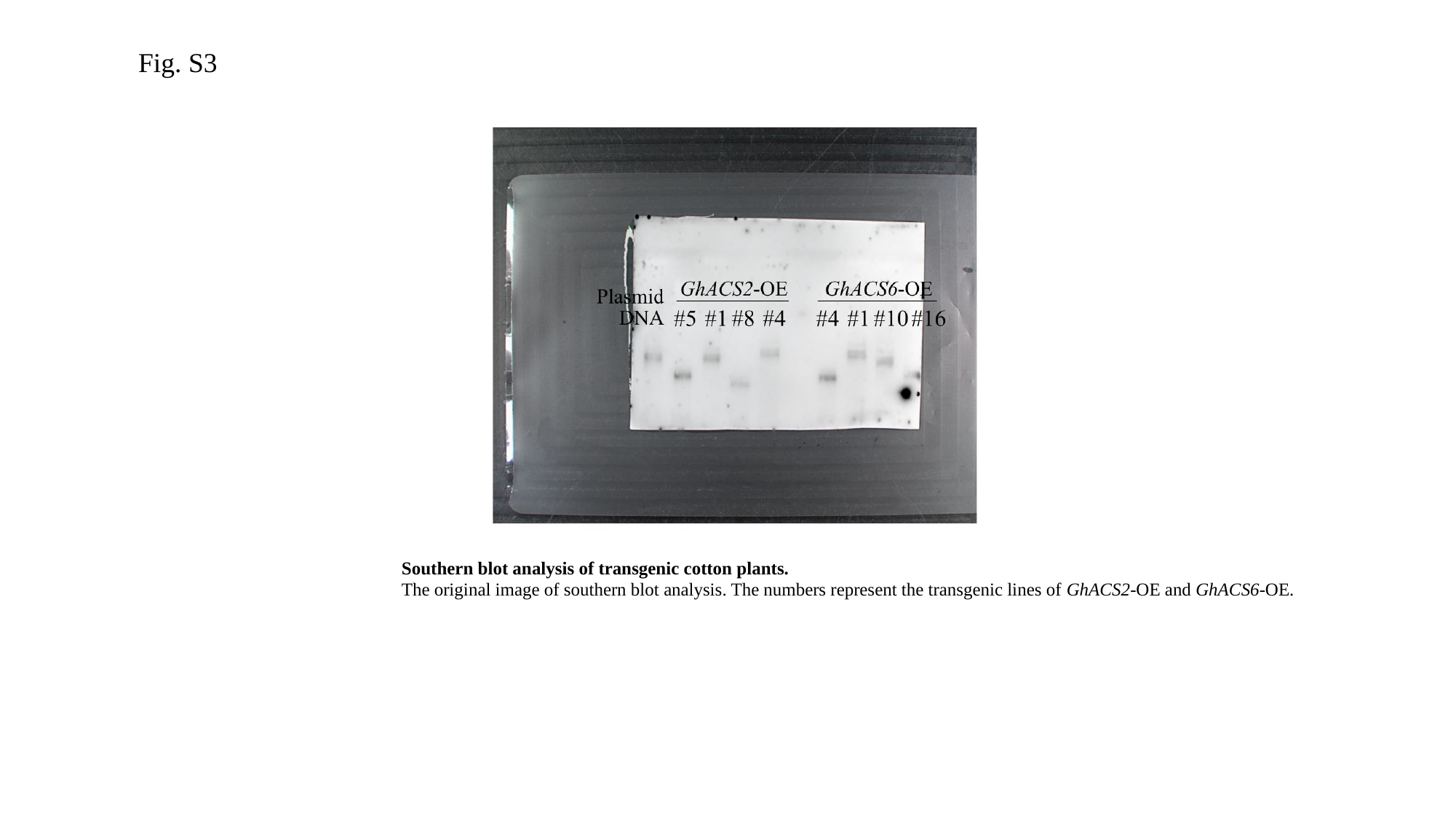

Fig. S3
Southern blot analysis of transgenic cotton plants.
The original image of southern blot analysis. The numbers represent the transgenic lines of GhACS2-OE and GhACS6-OE.

## Slide 4
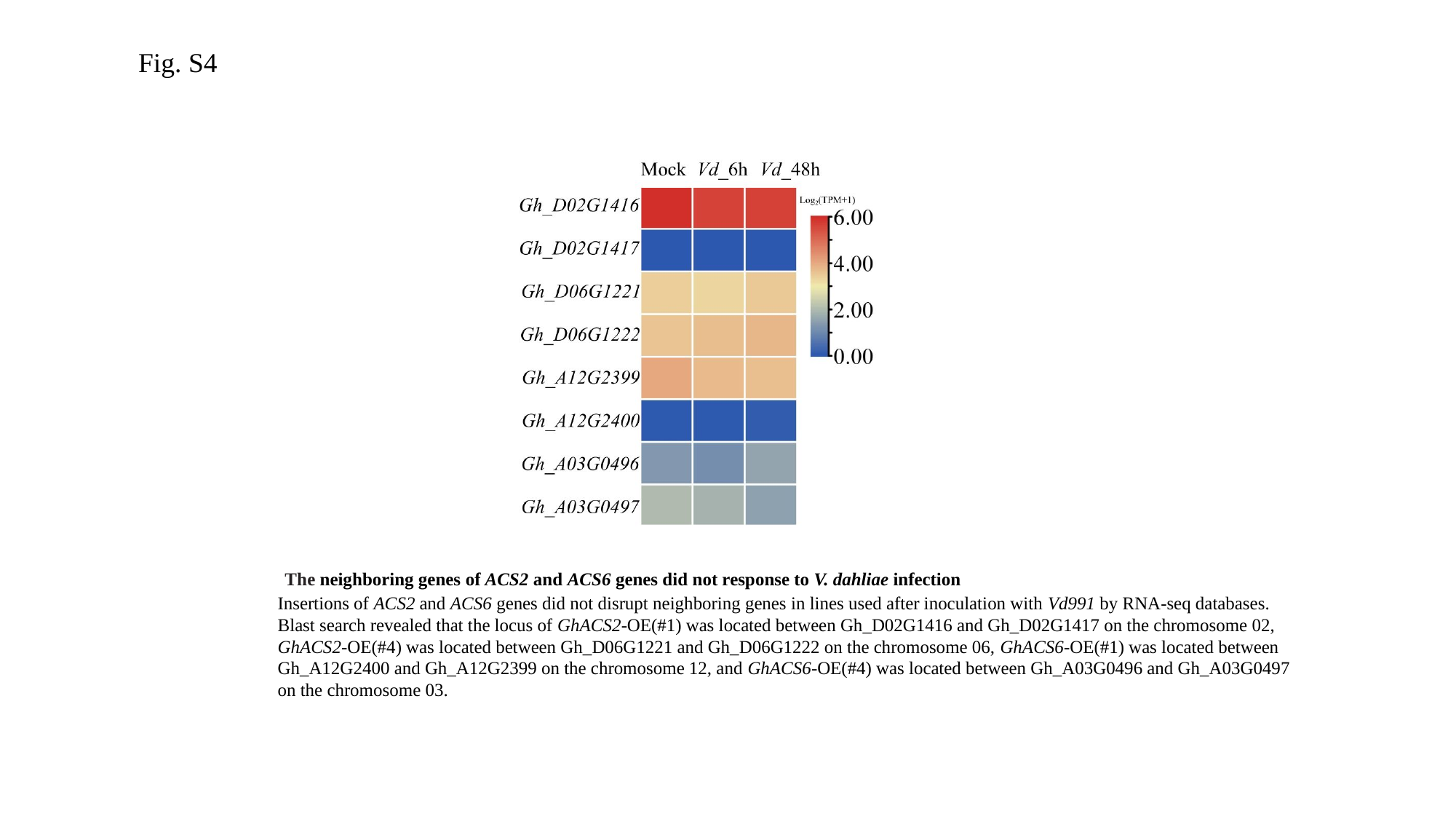

Fig. S4
 The neighboring genes of ACS2 and ACS6 genes did not response to V. dahliae infection
Insertions of ACS2 and ACS6 genes did not disrupt neighboring genes in lines used after inoculation with Vd991 by RNA-seq databases.
Blast search revealed that the locus of GhACS2-OE(#1) was located between Gh_D02G1416 and Gh_D02G1417 on the chromosome 02, GhACS2-OE(#4) was located between Gh_D06G1221 and Gh_D06G1222 on the chromosome 06, GhACS6-OE(#1) was located between Gh_A12G2400 and Gh_A12G2399 on the chromosome 12, and GhACS6-OE(#4) was located between Gh_A03G0496 and Gh_A03G0497 on the chromosome 03.

## Slide 5
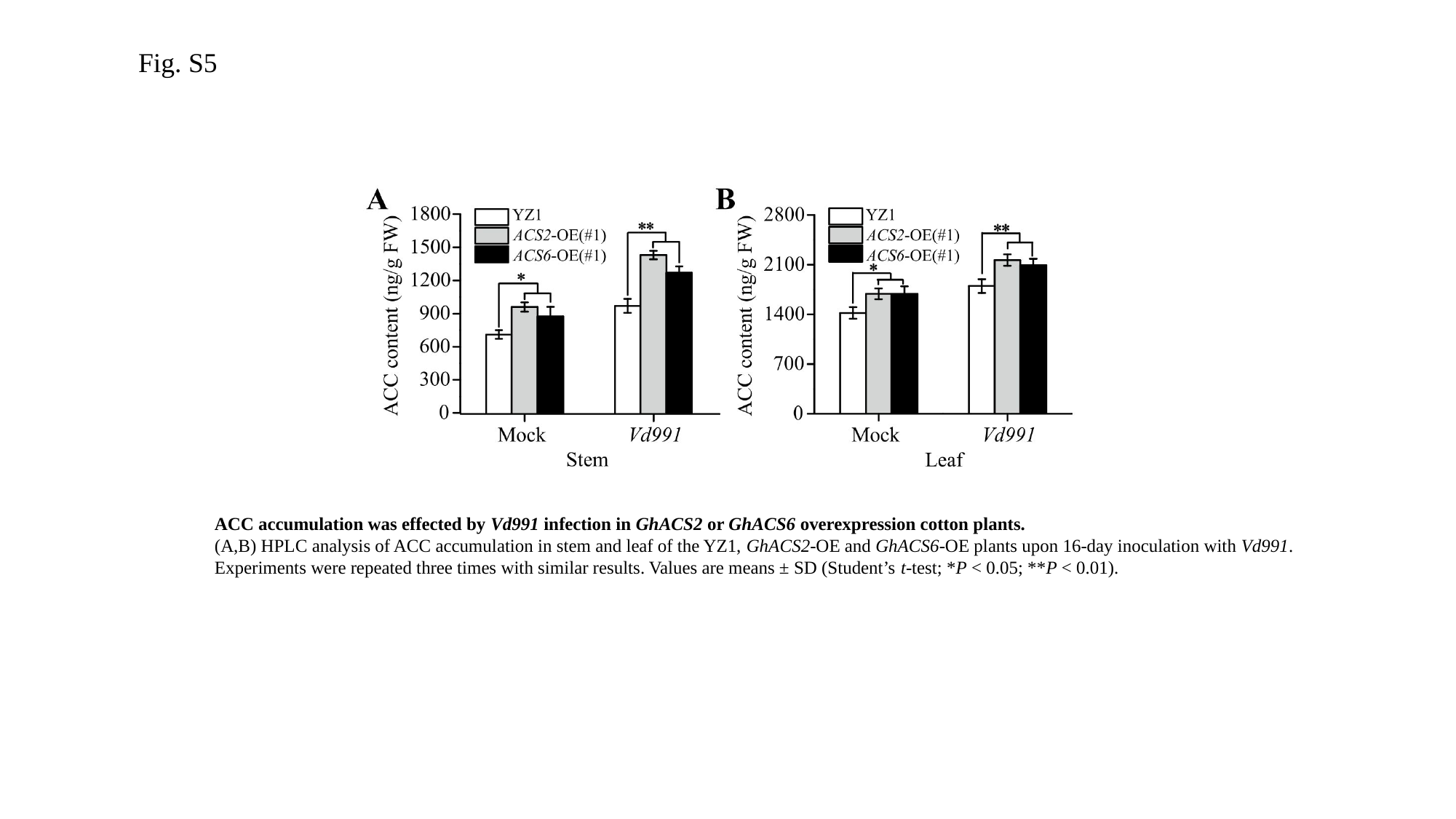

Fig. S5
ACC accumulation was effected by Vd991 infection in GhACS2 or GhACS6 overexpression cotton plants.
(A,B) HPLC analysis of ACC accumulation in stem and leaf of the YZ1, GhACS2-OE and GhACS6-OE plants upon 16-day inoculation with Vd991.
Experiments were repeated three times with similar results. Values are means ± SD (Student’s t-test; *P < 0.05; **P < 0.01).

## Slide 6
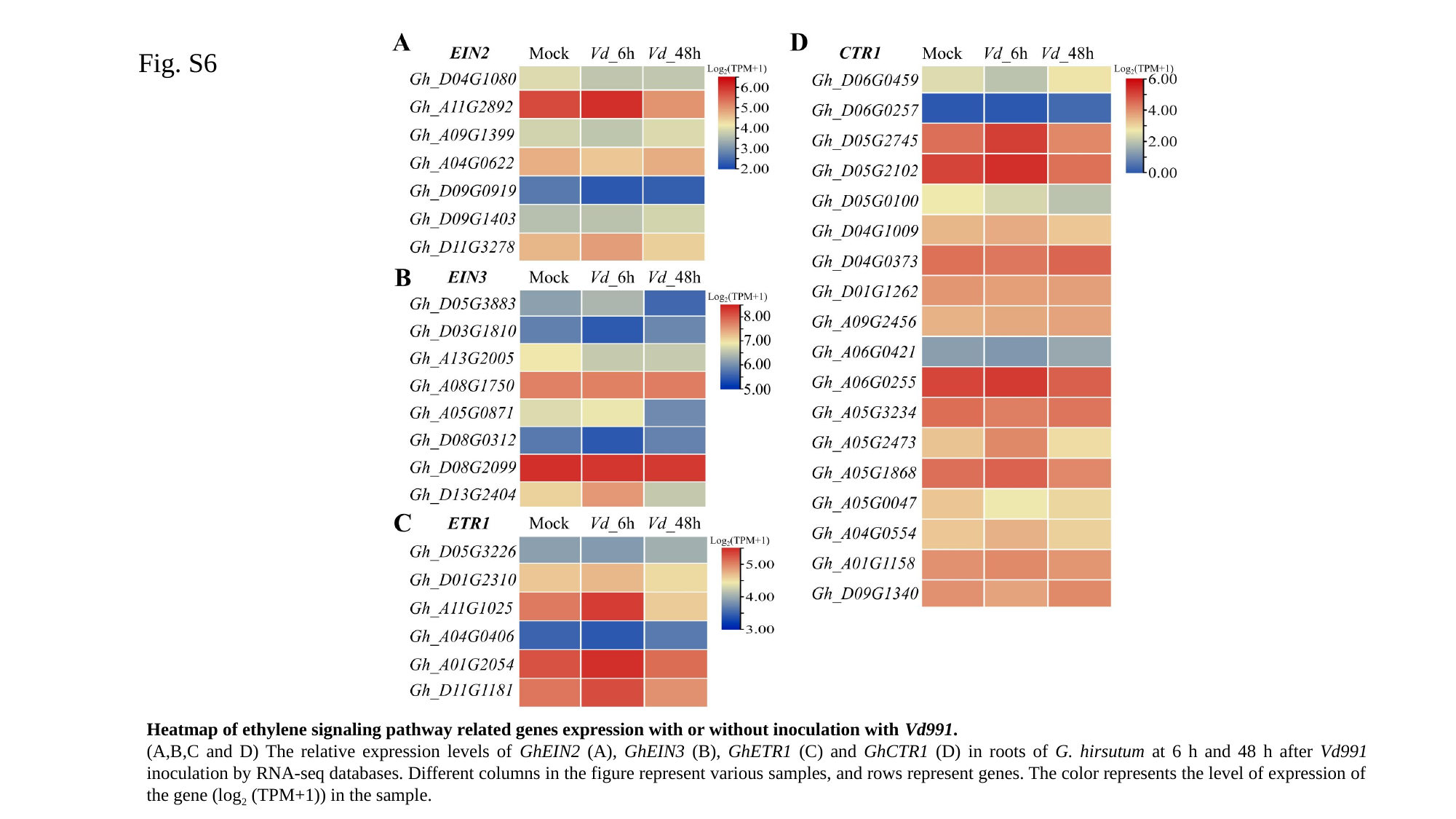

Fig. S6
Heatmap of ethylene signaling pathway related genes expression with or without inoculation with Vd991.
(A,B,C and D) The relative expression levels of GhEIN2 (A), GhEIN3 (B), GhETR1 (C) and GhCTR1 (D) in roots of G. hirsutum at 6 h and 48 h after Vd991 inoculation by RNA-seq databases. Different columns in the figure represent various samples, and rows represent genes. The color represents the level of expression of the gene (log2 (TPM+1)) in the sample.

## Slide 7
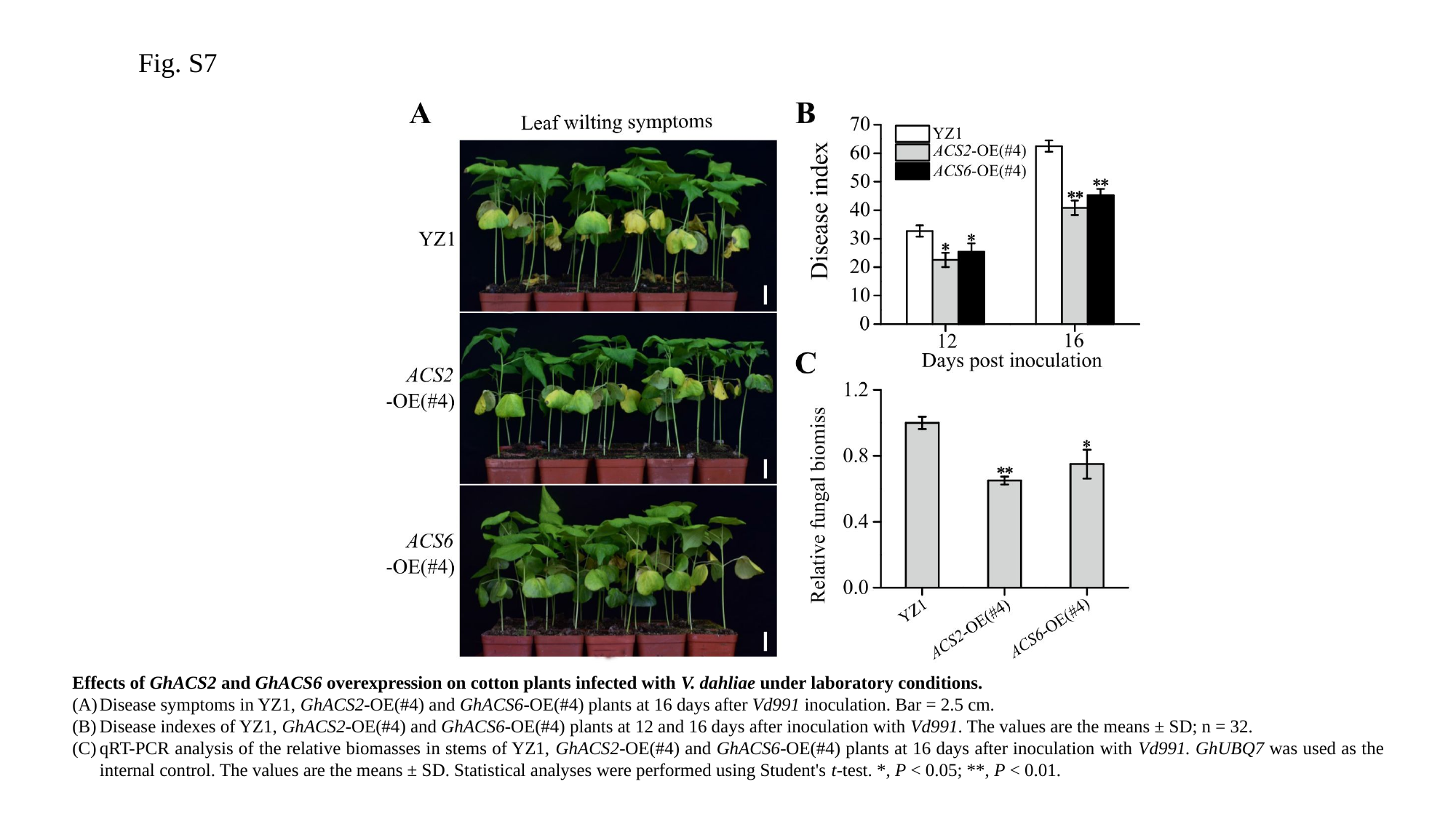

Fig. S7
Effects of GhACS2 and GhACS6 overexpression on cotton plants infected with V. dahliae under laboratory conditions.
Disease symptoms in YZ1, GhACS2-OE(#4) and GhACS6-OE(#4) plants at 16 days after Vd991 inoculation. Bar = 2.5 cm.
Disease indexes of YZ1, GhACS2-OE(#4) and GhACS6-OE(#4) plants at 12 and 16 days after inoculation with Vd991. The values are the means ± SD; n = 32.
qRT-PCR analysis of the relative biomasses in stems of YZ1, GhACS2-OE(#4) and GhACS6-OE(#4) plants at 16 days after inoculation with Vd991. GhUBQ7 was used as the internal control. The values are the means ± SD. Statistical analyses were performed using Student's t-test. *, P < 0.05; **, P < 0.01.

## Slide 8
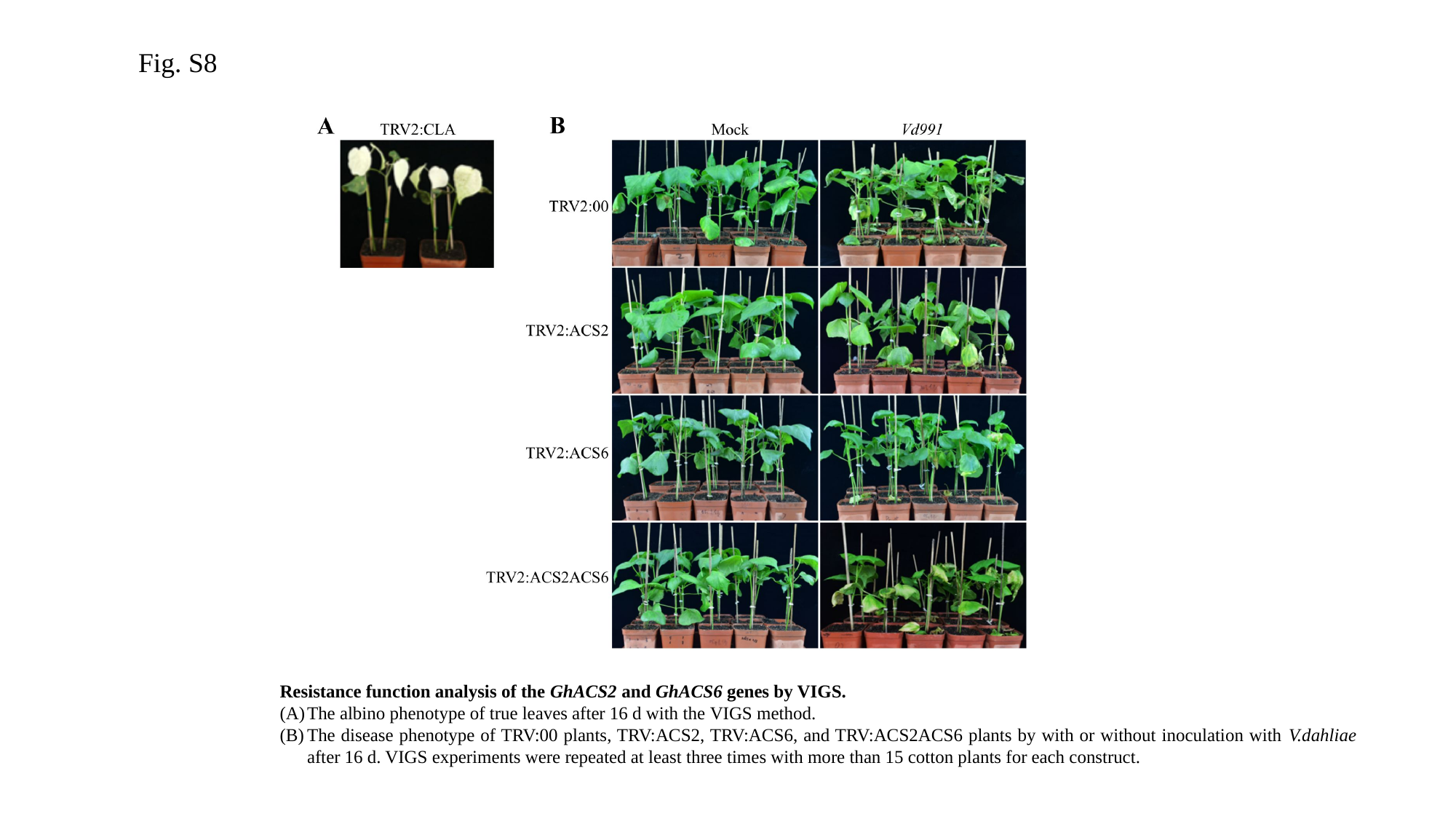

Fig. S8
Resistance function analysis of the GhACS2 and GhACS6 genes by VIGS.
The albino phenotype of true leaves after 16 d with the VIGS method.
The disease phenotype of TRV:00 plants, TRV:ACS2, TRV:ACS6, and TRV:ACS2ACS6 plants by with or without inoculation with V.dahliae after 16 d. VIGS experiments were repeated at least three times with more than 15 cotton plants for each construct.

## Slide 9
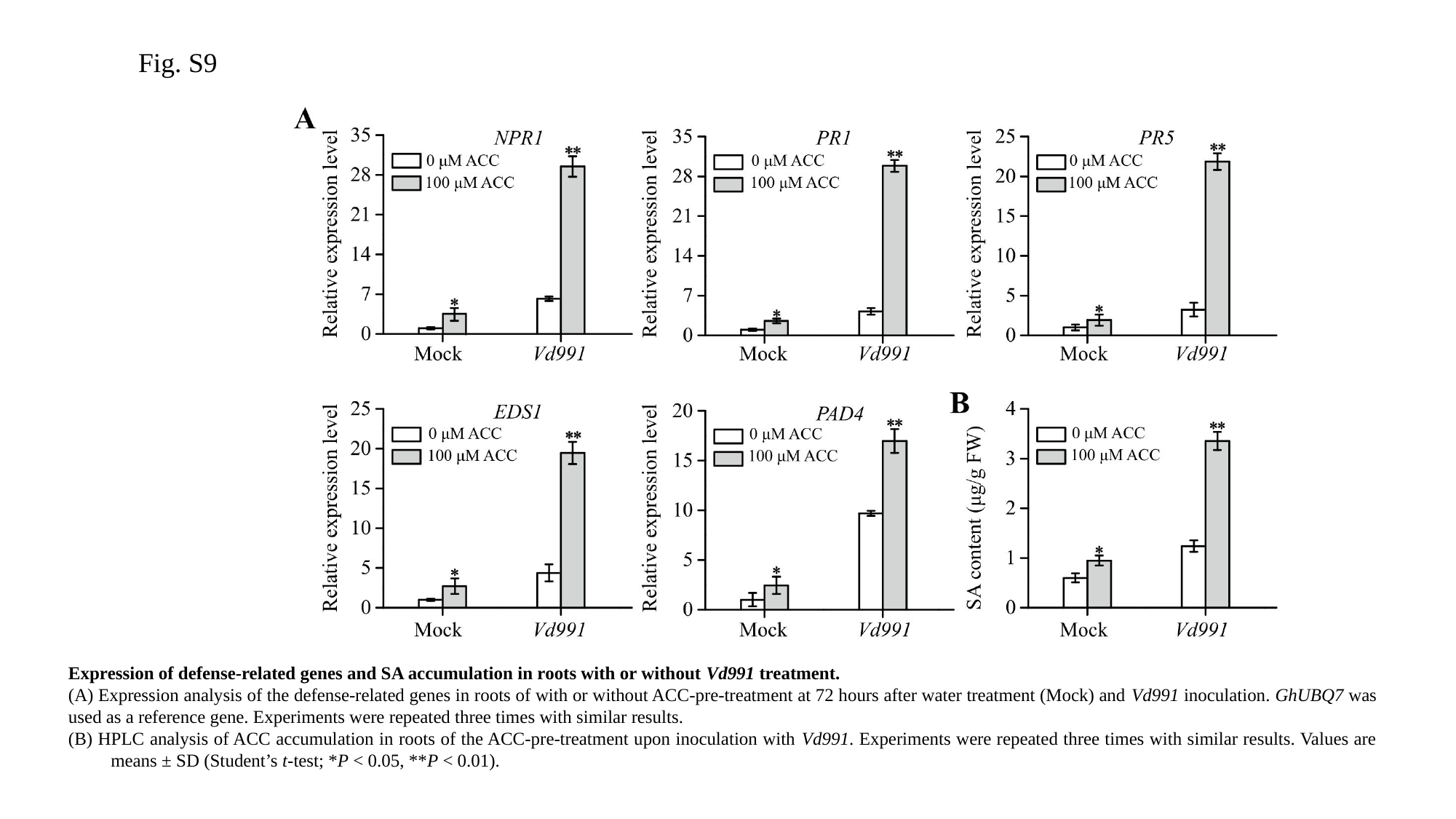

Fig. S9
Expression of defense-related genes and SA accumulation in roots with or without Vd991 treatment.
(A) Expression analysis of the defense-related genes in roots of with or without ACC-pre-treatment at 72 hours after water treatment (Mock) and Vd991 inoculation. GhUBQ7 was used as a reference gene. Experiments were repeated three times with similar results.
(B) HPLC analysis of ACC accumulation in roots of the ACC-pre-treatment upon inoculation with Vd991. Experiments were repeated three times with similar results. Values are means ± SD (Student’s t-test; *P < 0.05, **P < 0.01).
